# Development of a medication literacy assessment scale for patients with mental disorders in recovery in China: a mixed-methods Delphi study

**DOI:** 10.3389/fpsyt.2025.1551160

**Published:** 2025-05-27

**Authors:** Lin Deng, Bei Wu, Jianlin Lou, Chen Zhang, An Huang, An Gu, Haiqin Chen, Lina Wang

**Affiliations:** ^1^ School of Medicine, Huzhou University, Huzhou, Zhejiang, China; ^2^ Rory Meyers College of Nursing, New York University, New York, NY, United States; ^3^ The Outpatient Department of the First Affiliated Hospital, Huzhou University, Huzhou, Zhejiang, China; ^4^ Department of General Medicine, Community Health Service Center of Binhu Street, Huzhou, Zhejiang, China; ^5^ The Nursing Department of the Third People's Hospital of Huzhou, Huzhou, China

**Keywords:** mental disorders, medication literacy, recovery period, scale, assessment tools

## Abstract

**Background:**

Patients with mental disorders often exhibit unique challenges in medication adherence, comprehension of drug information, and self-management abilities, underscoring the need for a specialized assessment tool to accurately reflect their medication literacy levels and support targeted clinical interventions.

**Methods:**

A stepwise, mixed-methods design was adopted to develop the Medication Literacy Assessment Scale. Preliminary items were generated through a comprehensive literature review and semi-structured interviews with 20 patients and 6 psychiatric professionals. Then, a two-round Delphi study was conducted to refine the scale based on expert consensus. Quantitative analysis of expert feedback guided the scale’s refinement, ensuring it effectively captures the unique aspects of medication literacy for patients with mental disorders in recovery.

**Results:**

The finalized Medication Literacy Assessment Scale for patients with mental disorders in recovery was developed, yielding 35 items across four dimensions: functional literacy (10 items), communicative literacy (6 items), critical literacy (11 items), and numeracy (8 items). Each dimension reflects essential aspects of medication literacy specific to this population, as identified through expert consensus.

**Conclusion:**

This study developed a preliminary, standardized tool for assessing medication literacy in patients with mental disorders during recovery, with the potential to identify individuals at risk of medication mismanagement and to enable targeted interventions and improved outcomes in China’s healthcare system. Although its psychometric properties have not yet been evaluated in this stage, future research will conduct empirical validation to establish its measurement reliability and validity.

## 1 Introduction

Medication safety is a global health priority emphasized by the WHO’s ‘Medication Without Harm’ initiative, which targets reductions in medication-related harm through improved safety practices and increased patient medication literacy (1). Similarly, The National Health Service Improvement and NHS England United have recognized medication safety and mental health as key focus areas in their patient safety strategy (2). Furthermore, The National Health Commission of China has highlighted the critical importance of enhancing medication safety management, emphasizing the need for fundamental interventions such as medication explanations and reminders, alongside strengthened safety measures for specific drug categories, including high-risk, highly toxic, narcotic, and psychotropic medications (3). In psychiatric care, educating patients on the safe use of psychotropic medications is vital to ensure adherence and reduce the risk of relapse, particularly given the challenges of managing these medications outside clinical settings.

Medication literacy, as defined by Pouliot (4), involves the capacity to obtain, understand, and apply medication-related information for safe decision-making. It encompasses a range of competencies, including comprehension, communication, calculation, and information processing across various formats. Building on this foundational framework, Pantuzza developed a conceptual model of medication literacy that identifies four core clusters: functional literacy, communicative literacy, critical literacy, and numeracy. These clusters are essential for medication literacy and include competencies such as accessing reliable information, understanding medication effects and side effects, calculating dosages, and managing follow-up schedules (5). Previous studies on medication literacy have largely focused on patients with chronic physical conditions, such as diabetes, cardiovascular disease, and asthma, where inadequate medication literacy is closely linked to non-adherence, poor disease management, and increased healthcare costs (6–8). Medication adherence is a critical concern among patients with mental disorders, particularly during the recovery phase. However, research shows that many patients exhibit low levels of medication literacy, which undermines their ability to use medications safely and effectively. Whether in inpatient or outpatient settings, a substantial proportion of patients cannot correctly identify their psychotropic medications by name or type (9). Lau et al. (10) found that 26.8% of patients were unaware of their medication dosage, leading to dosing errors and misunderstanding about side effects and efficacy. Additionally, one study noted that patients with shorter illness durations were more likely to discontinue medication prematurely, while those with longer-term treatment demonstrated a better understanding of their medications (11). Non-adherence to prescribed medication regimens may increase the risk of relapse, self-harm, aggression, and poor quality of life (12, 13). Furthermore, patients with mental disorders often display problematic medication-related behaviors. Semahegn et al. showed that nearly half of psychiatric patients exhibit non-adherence, largely due to poor insight, adverse side effects, or misconceptions about the necessity and effectiveness of their medication (14). Some patients believe their condition will improve without medication or discontinue treatment once symptoms subside (15). Additionally, some patients lack insight into their illness, leading to denial, refusal of medication, or non-adherence to treatment plans (16). These behaviors significantly increase the risk of relapse, violence, social dysfunction, and reduced quality of life (12, 17). Research suggests (15) that these problematic medication behaviors are primarily rooted in a lack of scientific understanding of psychopharmacotherapy, compounded by insufficient awareness of the severity of mental disorders and the importance of correctly using psychiatric medications. Evidence indicates that early education on psychotropic medications, including their type, purpose, side effects, and duration, could improve both medication adherence and patient confidence in treatment (18). Therefore, it is necessary for psychiatric professionals to assess medication literacy early in pharmacological treatment and to monitor it continuously throughout long-term care. This approach could support patients gain accurate knowledge and understanding of their medications, potentially enabling safer and more appropriate medications use.

However, previous research on medication use among patients with mental disorders has largely focused on specific aspects rather than providing a comprehensive assessment of medication literacy. Tools like the Patient Satisfaction with Psychotropic scale assess patient attitudes toward side effects, treatment, and symptom relief (19), while others target single dimensions, such as the Knowledge about Schizophrenia Questionnaire (20). Similarly, disorder-specific tools, like the Anxiety Literacy Questionnaire (21) and the Depression Literacy Scale (22), measure knowledge within individual conditions. Although several medication literacy assessments have been designed for the general population (23–25), none are specifically tailored to the unique needs of patients with mental disorders, highlighting a gap in comprehensive assessment tools.

Given the distinct cognitive and behavioral challenges faced by patients with mental disorders and the complex nature of psychotropic medication regimens, this study aims to develop a tool specifically designed to assess medication literacy in patients with mental disorders in recovery, to accurately and objectively reflect their medication literacy levels. Based on the assessment results, personalized education and support will be provided to improve their medication literacy, thereby enhancing treatment outcomes, reducing side effects, and promoting recovery. Additionally, this tool will provide a theoretical foundation and practical resource for future research on the factors associated with medication literacy in the mental disorder population.

## 2 Methods

The instrument was developed using a two-phase, mixed-methods approach, involving (a) initial development of a draft medication literacy assessment scale for patients with mental disorders in recovery, based on data from a literature review and qualitative interviews; and (b) refinement of the scale through the Delphi method to reach expert consensus. The development process of the scale was guided by select criteria from the Consensus-based Standards for the Selection of Health Measurement Instruments (COSMIN) checklist (26), ensuring a rigorous evaluation focused on key aspects of measurement quality. This scale is designed to support psychiatric professionals in quantifying medication literacy levels and identifying individuals at higher risk for mismanagement. By enhancing medication safety and supporting targeted interventions, this scale may also serve as a foundation for future research on medication literacy in mental health care.

### 2.1 Establishment of subject panels

The panel consisted of nine members: one professor of mental health nursing, two chief psychiatrists, two chief psychiatric nurse practitioners, one chief pharmacist specializing in psychiatry, and three registered mental health nurses. The panel’s primary tasks included developing preliminary scale dimensions and item pools through a literature review, refining item pools through iterative discussions, designing expert consultation questionnaires, and analyzing feedback from the expert consultations.

### 2.2 Phase 1 of scale development

#### 2.2.1 Literature review

This study systematically searched the Web of Science, PubMed, Elsevier ScienceDirect, Embase, Cochrane, China National Knowledge Infrastructure, and Wan Fang databases for Chinese and English literature on medication literacy in patients with mental disorders from their inception to December 2023. Keywords included “medication literacy”, “pharmacotherapy literacy”, “Pharmacy literacy”, “drug literacy”, “medication information literacy”, “medication health literacy”, and “medication knowledge and literacy”. Due to limited research on medication literacy in patients with mental disorders, the search terms were not confined to a specific population. Based on the definition and conceptual models of medication literacy, along with relevant guidelines for mental disorders treatment and management (27–29), an initial framework and item pool for the scale were developed.

For Data extraction, synthesis and item generation, the literature review systematically organized existing research on medication literacy, including relevant theoretical frameworks, measurement tools, and relevant scales. This review provided a foundation for the subsequent Delphi process, ensuring that the initial items and dimensions were grounded in existing evidence. Based on this review, a preliminary item pool was developed, covering key dimensions of medication literacy across different contexts, and served as the basis for expert evaluation and refinement during the Delphi rounds.

#### 2.2.2 Semi-structured interviews

A semi-structured interview outline was developed, and interviews were conducted with patients in recovery from mental disorders and psychiatric professionals (psychiatrists and psychiatric nurses) between March and May 2024 to validate and enhance the initial scale items. Participants were recruited through convenience sampling from the Third Municipal Hospital, Huzhou, China.

(1) Inclusion criteria for patients were: (a) age ≥18 years and meeting ICD-10 diagnostic criteria for schizophrenia, schizoaffective disorder, paranoid psychosis, epilepsy-induced psychotic disorder, depressive disorder, bipolar disorder, and anxiety disorder; (b) completion of acute and consolidation treatment stages, with a Positive and Negative Symptom Scale (PANSS) total score reduction rate of ≥ 50% or a total PANSS score ≤ 60, indicating stability with controlled hallucinations, delusions, improved mood, and normalized behavior diagnosed by the doctor; (c) absence of visual or hearing impairments; (d) clear consciousness, and ability to understand and communicate verbally; (e) voluntary participation with informed consent. Exclusion criteria included current or past diagnoses of delirium, dementia, intellectual disability, mental disorders due to somatic conditions or psychoactive substances, serious and unstable physical illnesses, a history of traumatic brain injury or other known organic central nervous system disease, or an elevated risk of suicide or violence.

(2) Inclusion criteria for psychiatric professionals were: (a) ≥ 7 years of clinical experience in psychiatry or recognized expertise in the field; (b) intermediate or higher professional title; (c) bachelor’s degree or higher; and (d) voluntary participation. Exclusion criteria included trainees, nurses or physicians temporarily assigned to psychiatry from other medical departments, and administrators.

Patients were recruited from hospital through clinician referrals, while psychiatric professionals were purposively selected based on their clinical experience in medication management. All participants were approached in person, informed about the study purpose and procedures, and provided written informed consent prior to participation. The semi-structured interview is conducted face-to-face. The interview was conducted by one researcher, who had received qualitative research training. Interviews were conducted in a quiet, soundproof room and scheduled at the participants’ convenience to minimize distractions. The process followed the principles of sample adequacy and data saturation.

During data collections, interviews and observations were used in combination. A detailed interview outlines are shown in **Table 1**. Each interview lasted approximately 15 to 30 minutes, and was audio-recorded with the interviewee’s consent. Meanwhile, non-verbal behaviors such as facial expressions and body language were also documented. Interviews were concluded once information saturation was reached. After the interview, within 24 hours, two researchers anonymized and coded the data. Patient identifiers began with “P” (e.g., P1, P2), while psychiatric professionals were labeled with “M” (e.g., M1, M2). All recordings were transcribed verbatim, and transcripts were cross-checked for accuracy and completeness.

**Table 1:** Interviews outline for patients and psychiatric professionals.

Interviewees	Outline of an interview
Patients with mental disorder	1. What role do you believe adherence to psychotropic medication plays in the treatment of mental disorders?
	2. Please describe your knowledge of the medication you are currently prescribed.
	3. What concerns or difficulties do you experience with your current medication?
	4. How do you typically resolve any challenges or confusion related to your medication?
	5. What additional needs do you have in managing your medication?
Psychiatric professionals	1. What common medication-related issues do you observe among patients with mental disorders in recovery?
	2. What aspects of psychotropic medications do patients most frequently ask about?
	3. In your opinion, what information, knowledge, or skills should patients possess regarding psychotropic medications?
	4. What key points do you emphasize when educating patients with mental disorders about psychotropic medications?

Data organization and analysis were conducted as follows: Qualitative data were analyzed using content analysis (30). Two researchers independently performed the coding. Transcripts were repeatedly reviewed to achieve a comprehensive understanding, followed by line-by-line coding to extract significant statements. Recurring statements were grouped into themes, and thematic relationships across transcripts were examined to form thematic clusters. Data collection and analysis proceeded concurrently until saturation was reached, identifying key items related to medication literacy. NVivo 11 (QSR) software was used to support data coding and organization.

#### 2.2.3 Integration of literature review and interviews

The initial item pool for the Delphi process was developed by combining findings from a focused literature review and semi-structured interviews with patients in recovery and psychiatric professionals. The literature helped establish the theoretical structure of the scale, while the interviews offered practical insights into how medication is understood and used in clinical settings. Key themes from both sources were used to generate draft items for the first-round Delphi questionnaire.

### 2.3 Phase 2 of scale development

The Delphi method was used in this study, following the Conducting and Reporting of Delphi Studies (CREDES) guideline (31), The CREDES checklist for this study is provided in **Supplementary Material 2**. The COSMIN standards for evaluating the quality of PROM development were used as guidelines for developing the measurement tools, as detailed in **Supplementary Material 3**.

#### 2.3.1 Sample

A purposive sample of 15 clinical and nursing experts in psychiatry was selected. With extensive experience in the care of patients with mental disorders during recovery, these experts provided valuable insights into medication management and literacy. Their involvement ensured that the assessment scale was clinically relevant, practical, and aligned with patients’ real-world needs. Based on the study objectives and the effective response rate of the expert consultation, 2 rounds of consultation were performed (32). Experts met the following inclusion criteria: (a) medical or nursing specialists with over 10 years of experience or a recognized academic standing in psychiatric research or clinical practice; (b) intermediate or higher professional title; (c) bachelor’s degree or higher; and (d) voluntary participation.

#### 2.3.2 Development and distribution of consultation questionnaires

The expert consultation consisted of four sections (1): Introduction, providing the study’s purpose, significance, theoretical basis, relevant assessment concepts, and instructions for questionnaire completion, including return timeline and method (2); Expert background information (3); Expert evaluation, was conducted using a 5-point Likert scale, ranging from ‘very important - 5’ to ‘not important at all - 1’, to rate the importance of each item. This scoring format was selected for its theoretical and practical advantages in balancing response differentiation and cognitive load (33), promoting consistency (34), and supporting Delphi’s iterative consensus process (32). Experts also evaluated item alignment with corresponding dimensions, language clarity, and relevance, offering suggestions for modification. An ‘Additional Indicators’ section allowed experts to propose new items, including ratings of their importance and rationale (4); Self-evaluation of expert authority, in which experts specified the basis for their ratings, rated as ‘High’, ‘Medium’, or ‘Low’. Familiarity with the content was rated from very familiar (1.0) to very unfamiliar (0.2). The Consultation questionnaires were piloted with five non-participants (a professor of mental health nursing, a chief psychiatrist, a chief psychiatric pharmacist, and two registered mental health nurses) to ensure clarity, relevance, and timeliness. Revisions were made as necessary.

**Figure 1:**
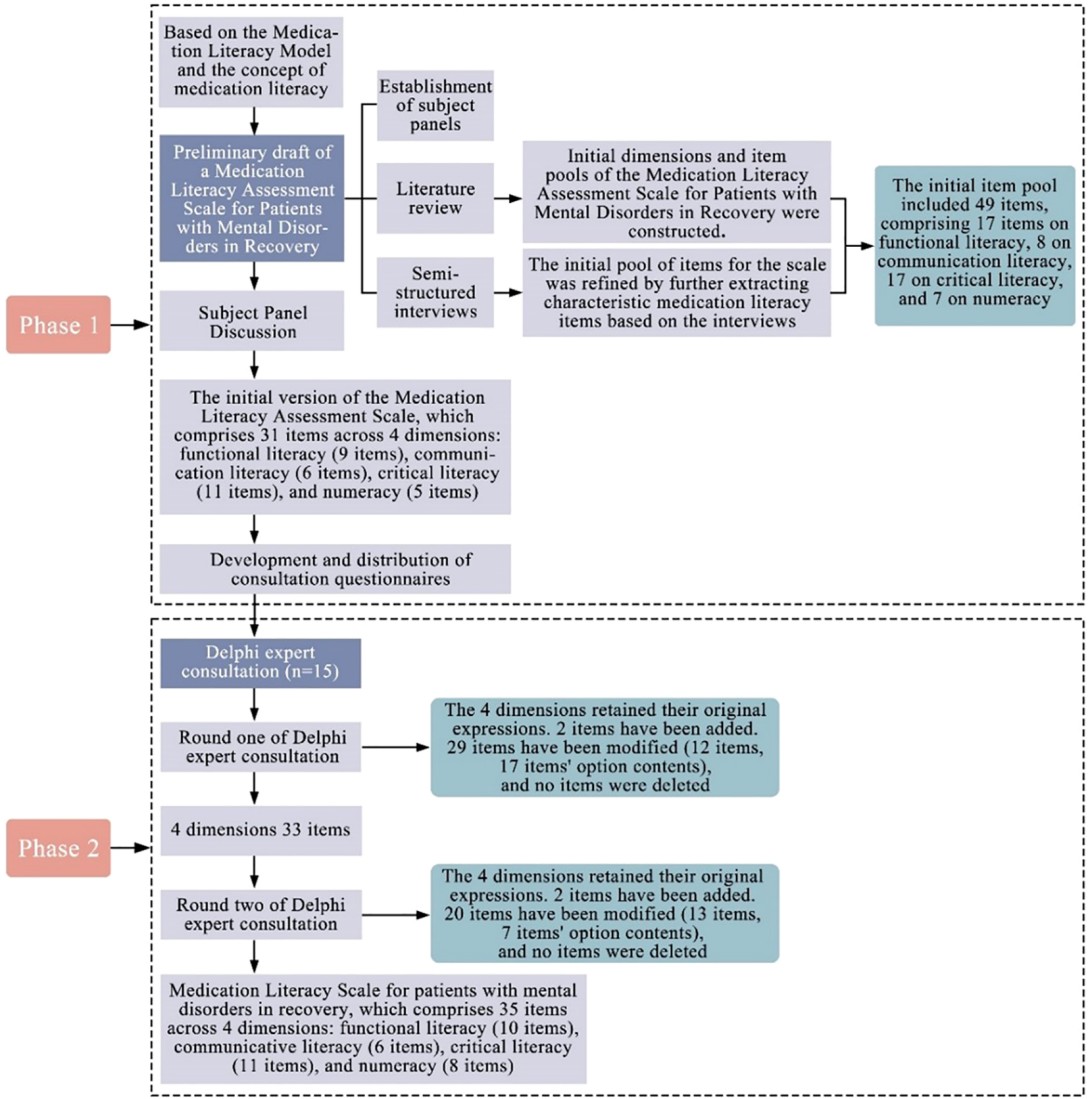
Flowchart for medication literacy assessment scale development.

With expert consent, the questionnaires were emailed individually with instructions to complete independently and return feedback within two weeks. A two-round Delphi method was used to refine the initial item pool. In the first round, experts were invited to evaluate each item in terms of importance, clarity, and relevance using a structured questionnaire. The collected responses were analyzed quantitatively to assess expert agreement. Based on these results and written feedback from the experts, items were reviewed for potential revision, deletion, or addition. The revised questionnaire was distributed to the same expert panel in the second round. Experts re-evaluated the modified items, and the same statistical approach was used to determine the level of consensus. Items that reached the predefined consensus criteria were retained in the final scale. The Delphi consultation process involved two rounds conducted from June to September 2024. **Figure 1** shows the development flowchart of the Medication Literacy Assessment Scale.

#### 2.3.3 Data analysis

Data analysis was performed using IBM SPSS 25.0. Descriptive statistics are reported as mean, standard deviation (SD), and coefficient of variation (Cv). The effectiveness of expert responses was assessed by the positive coefficient, defined as the questionnaire response rate, with a threshold of ≥ 70% indicating effective response (35). Expert authority was assessed using the expert authority coefficient (Cr), calculated from the familiarity coefficient (Cs) and the judgment coefficient (Ca) with the formula Cr = (Cs + Ca)/2, and Cr ≥ 0.7 is considered to reflect reliable input (36). The consensus among experts was measured using the coefficient of variation (Cv) and Kendall’s Coefficient of Concordance (Kendall’s W), with a Cv < 0.25 as the retention criterion per round (37). Kendall’s W was used to assess the degree of agreement (range: 0-1). Statistical significance was evaluated using a χ^2^ test, with p < 0.05 indicating significant consensus. The importance of each item was measured by the full score frequency (K), with > 20% deemed acceptable. Items were retained if they met the following criteria: full score frequency > 20%, mean significance rating > 3.5, and Cv < 0.25. Final decisions on item retention, modification, or deletion were made based on expert feedback and panel discussion.

#### 2.3.4 Ethics

Ethical approval was obtained from the Research Ethics Committee of Hu Zhou Third Municipal Hospital (approval number: 2024031) prior to data collection and registered at Chictr.org.cn (ChiCTR 2400091901). All participants were informed of the study’s purpose, procedures, and confidentiality protections and provided written informed consent.

## 3 Results

### 3.1 Results from the literature review

**Figure 2:**
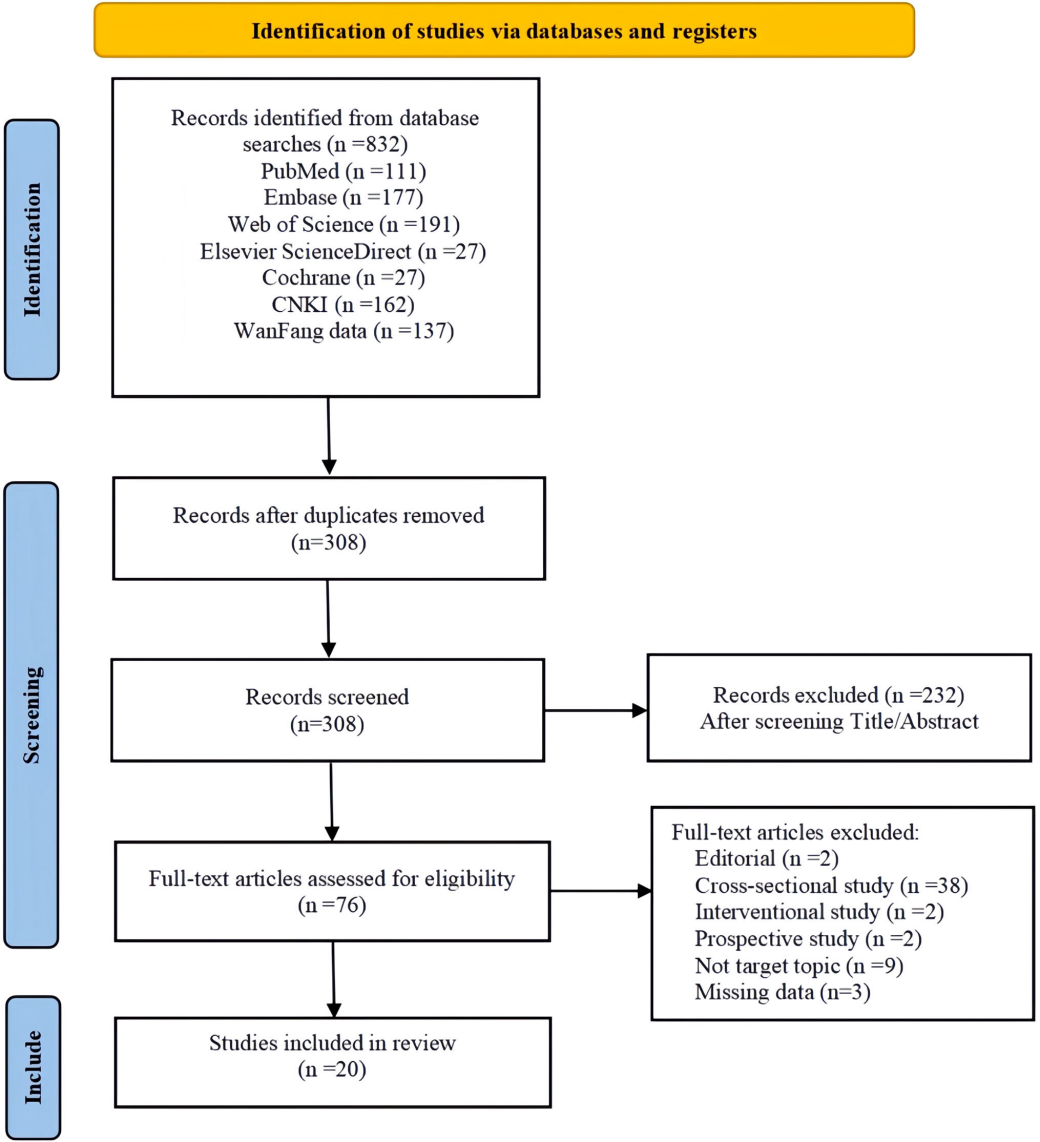
PRISMA flow diagram of study selection process.

This study systematically searched major domestic and international databases, resulting in a total of 832 articles. After removing duplicates using EndNote software, 308 articles remained. After reading the titles and abstracts, 232 articles were excluded. After reading the full text, 56 articles were excluded, leaving 20 articles for inclusion, including 15 psychometric studies, two cross-sectional studies, one qualitative methodology, one review study and one Delphi study. Additional characteristics of the included studies were shown in **Supplementary Material 4**. The literature search process is shown in **Figure 2**.

Previous studies have developed both general and population-specific tools to assess medication literacy. General tools are often one-dimensional; for example, the Medication Literacy Questionnaire by Maniaci assesses functional literacy by examining knowledge of medication name, quantity, dosage, administration timing, purpose, and potential adverse effects (38). Other general tools include the Medication Literacy Assessment in Spanish and English (Med Lit Rx SE) (23), the Medication Health Literacy Measure (39), and the Chinese Medication Literacy Measure (Ch MLM) (24). While Med Lit Rx SE addresses non-prescription use for pediatric fever and insulin use, making it suitable for diabetics and parents, it does not assess information access skills. Similarly, the Medication Health Literacy Measure focuses on label identification for diabetes and immunosuppressant medications, limiting its versatility. Ch MLM, which explores dietary supplement side effects as presented in advertisements, poses applicability challenges for its intended population.

Specialized tools, such as the Medication Literacy Scale for Caregivers of Patients with Schizophrenia (40), capture caregiver perspectives on schizophrenia patients’ medication use, which may not accurately reflect patients’ own medication literacy. Other targeted tools address specific populations, such as pregnant women (41), hemodialysis patients (42), and hypertensive individuals (43), or focus on age groups, including children, adolescents (44), and older adults (45). However, many of these instruments lack clear operational definitions of medication literacy and fail to capture essential dimensions, such as attitudes toward medication, self-management behaviors, social support, and self-knowledge, which are particularly relevant for patients with mental disorders.

This study developed an initial framework and item pool for medication literacy tailored to patients with mental disorders, informed by a comprehensive literature review and conceptual models of medication literacy (4, 5). The subject panels compiled and organized the items based on a review of medication literacy literature, guidelines and consensus on the diagnosis and management of mental disorders. Items 1-13, 15-31, and 40-49 were derived from the literature on medication literacy, while items 4, 10, 13-14, 24-25, and 32-39 were supported by clinical guidelines and expert consensus. This process resulted in an initial item pool of 49 items, detailed in **Supplementary Material 1**.

### 3.2 Results of semi-structured interviews with psychiatric professionals and patients with mental disorders in recovery

#### 3.2.1 Participant characteristics

The interview data reached information saturation after the 20th patient with mental disorders in recovery, while the interviews with psychiatric professionals reached saturation after the 6th participant. Therefore, a total of 20 patients with mental disorders in recovery and 6 psychiatric professionals participated in semi-structured interviews. More detailed information is shown in **Table 2**.

**Table 2:** Characteristics of participants engage in semi-structured interviews.

Items	Patients (N=20)	Items	Professionals (N=6)
	n (%)		n (%)
Age, mean ± SD	40.15 ± 13.18	Age, mean ± SD	37.83 ± 7.76
Gender		Gender	
Male	10 (50%)	Male	3 (50%)
Female	10 (50%)	Female	3 (50%)
Education level		Education level	
Primary and below	3 (15%)	Undergraduate	3 (50%)
Middle and high school	9 (45%)	Master	3 (50%)
Secondary and junior colleges	6 (30%)	Years of experience in psychiatry	
Bachelor’s degree or above	2 (10%)	7 ≤ Years < 10	4 (66.7%)
Years with mental disorders		10 ≤ Years < 20	1 (16.7%)
< 5 years	10 (50%)	Years ≥ 20	1 (16.7%)
5-10 years	4 (20%)	Psychiatry positions	
> 10 years	6 (30%)	Head nurse of psychiatry	2 (33.3%)
Type of medication		Psychiatrist	4 (66.7%)
1-2	10 (50%)	Professional title	
3-4	9 (45%)	Co-chief nurse	2 (33.3%)
≥ 5	1 (5%)	Attending physician	2 (33.3%)
Disease diagnosis		Associate chief physician	1 (16.7%)
Schizophrenia	6 (30%)	Chief physician	1 (16.7%)
Bipolar disorder	5 (25%)		
Depressive disorder	3 (15%)		
Anxiety disorder	6 (30%)		

#### 3.2.2 Qualitative content analysis

By analyzing the results of the interviews, significant statements were summarized as potential items for the scale, supporting item selection. Based on the medication literacy model (5), and the findings of literature review, and the semi-structured interviews, this study identified four primary themes: ‘functional literacy’, ‘communicative literacy’, ‘critical literacy’ and ‘numeracy’. **Table 3** presents the themes, subthemes, and representative quotes. The semi-structured interviews supplemented and validated items 3-4, 6-7, 11-15, 17-24, 26, 29-42, and 45-49, as detailed in **Supplementary Material 1**.

**Table 3:** Themes, subthemes, and representative quotes of the participants.

Themes	Subthemes	Representative quotes	
		Patients	Professionals
Functional literacy	- Concentrate on essential information related to current medication - Challenges in obtaining information - Insufficient initiative	P2-”Sometimes I just check the instructions and mainly look at the contraindications, not really paying attention to much else.” P8- “I’m trying to find better ways to get information about my medication, but I’m not sure where to start. Honestly, after taking it for so many years, I’ve kind of lost interest in figuring it out.” P11-”I’ve never really paid attention to medication information.” P14-”I didn’t pay much attention to medication information; I just trusted the hospital and didn’t actively keep track of it myself.”	M1- “Patients should understand their daily medication regimen, including how much they should take and what to look for on the medication labels.” M3- “Patients should familiarise themselves with the basic names of their medications, the dosages, and the effects they can expect.” M5- “They need to master the proper method of administration and know the duration the medication should be taken.” M6- “First, it’s important to know the appropriate timing for each medication—some are intended for morning use, while others are better suited for the evening. Taking medication at the wrong time can impact their daily routine. Moreover, patients should be aware of the common side effects associated with the psychotropic medications they are taking.”
Communicative literacy	- Methods of accessing medication information - Utilization of social support networks	P5- “I ask my parents about medications that I don’t understand, and I also consult via the internet.” P7- “When I have a follow-up appointment, the doctor sometimes prescribes me a new medication. If I’m not sure what it does, I ask the doctor right then and there.” P10- “I’ll ask my doctor about how long I need to stay on the medication.” P14- “I go online to read about other people’s experiences with the same medication—the challenges they faced and how they managed to overcome them and beat the illness. It really inspires me and gives me hope!”	M1- “The patient is interested in whether the medication has any effect on their body.” M2- “Some patients may have questions about why they’re prescribed certain medications. For example, someone with bipolar disorder might be prescribed valproic acid, a drug commonly used for epilepsy. This can lead to doubts, as they might think, “I don’t have epilepsy, so why am I taking this medication?” This kind of concern is common among patients who are prescribed anti-epileptic medications for other conditions.” M3- “One of the most common questions patients ask is, ‘What effect will this medication have? Why am I being prescribed this?”
Critical literacy	- Side effects of medication impacting daily life - Management of medication side effects - Handling common situations during medication use - Mistakes in medication-related decision-making - Recognition of early warning signs of disease	P5- “I had hand tremors, memory loss, trouble remembering things, felt sleepy, had no energy, and didn’t know what to do after taking the medication.” P6- “I’ve been on medication for over a year, but I felt like I didn’t need it anymore. I didn’t want to take it, so I stopped on my own and ended up relapsing.” P9- “Sometimes I suddenly forget to take my medication and only remember at noon or in the evening, and I don’t know if I’m still taking it or not.” P11- “I’ll suddenly get in a really bad mood and feel like I want to die, so I take a few extra pills. Then I regret it and don’t know how to handle it.” P12- “I’ve gained weight on the medication, and it’s hard to lose. My appetite has gone up, I get sleepy, and I’m less productive. I don’t know what to do about it.”	M1- “Patients taking clozapine often experience postural hypotension, like feeling dizzy when they get up in the morning. If they’re at home and this happens, they need to know how to handle it. Some meds also cause constipation or stomach discomfort, and patients should know basic ways to manage these issues. They’re not always able to reach a doctor or someone right away, so having some simple self-care skills for situations like these is essential.” M2- “For patients with recurring symptoms, there are times they might suddenly have negative thoughts and take a lot of medication impulsively, then regret it shortly after. They should know how to respond quickly, like inducing vomiting if needed. It’s really important they learn skills like this to help themselves in these situations.” M5- “Patients should learn to recognize the early warning signs of their illness. If their symptoms aren’t fully controlled by medication and they start noticing signs of a relapse, they should see a doctor as soon as possible, let their family know, and make arrangements to get to the hospital for a follow-up check.”
Numeracy	- Checking medication expiration dates - Scheduling follow-up appointments - Calculating medication duration	P4- “I don’t know the exact expiration date of my medication.” P8- “I can never find the expiration date on the top of the medicine box, and when I do, sometimes I don’t know how to read it.” P9- “After each prescription, I’m not sure how long I should take it before going back to the hospital for a follow-up.” P13- “I don’t know how many days the rest of the medication will last.”	M3- “Patients should know when their next follow-up is and make sure to attend regular check-ups. This way, the doctor can keep track of their condition, monitor any side effects, adjust medications if needed, and provide other helpful recommendations.” M5- “Patients need to keep track of how much medication they have left and when they need to get it refilled. This ensures they don’t run out and can stay on track with their treatment.”

Patients were labelled with ‘P’ (e.g., P1), and psychiatric professionals with ‘M’ (e.g., M1).

### 3.3 Panel discussion

Drawing on the insights gained from the literature review and semi-structured interviews, the constructs and dimensions of the Medication Literacy Assessment Scale for patients with mental disorders in recovery were initially established. **Table 4** presents the dimensions and connotations of medication literacy specific to patients with mental disorders in recovery. This initial pool includes 17 items on functional literacy, 8 on communication literacy, 17 on critical literacy, and 7 on numeracy, with details provided in **Supplementary Material 1**. To ensure the relevance and accuracy of the scale’s items, subject panels then integrated considerations of the unique characteristics of mental disorders, the specific properties of psychotropic medications, and patients’ medication adherence behaviors. The initial expert-reviewed version of the Medication Literacy Assessment Scale was developed, which comprises 31 items across 4 dimensions: functional literacy (9 items), communication literacy (6 items), critical literacy (11 items), and numeracy (5 items). Each item is scored using a 5-point Likert scale (1–5), with reference options available to assist patient responses. The details have been shown in **Supplementary Material 1**.

**Table 4:** Dimensions and connotations of medication literacy for patients with mental disorders in recovery.

Dimension	Connotations
Functional literacy	Encompasses the fundamental ability to understand essential information regarding prescribed psychotropic medications, including the medication’s name, purpose, dosage instructions, potential effects, and precautions. This dimension highlights the patient’s capacity to interpret and effectively apply this information.
Communicative literacy	Refers to the ability of patients with mental disorders to effectively access and convey information regarding psychotropic medications. This includes locating and understanding relevant information from various sources and effectively communicating medication-related concerns to healthcare professionals through verbal, written, or non-verbal cues.
Critical literacy	Includes the skills required for patients with mental disorders to critically evaluate, interpret, and judge information about psychotropic medications from various sources. This dimension also involves the capacity to effectively respond to emergencies that may arise during the use of these medications.
Numeracy	Involves the ability of individuals with mental disorders to perform calculations related to psychotropic medications, including addition, subtraction, multiplication, and division, based on information obtained from various sources.

### 3.4 Results of expert consultation

#### 3.4.1 Basic information on experts

Fifteen experts from China participated in this study, contributing to two rounds of the Delphi process. The experts had a mean age of 44.6 ± 10.16 years, with 66.67% being women and 33.33% holding doctoral qualifications. Their experience in the psychiatry field averaged 18.73 ± 11.8 years. Detailed demographics are outlined in **Table 5**.

**Table 5:** The basic information on experts (n=15).

Characteristics	N (%)	
Gender		
Male	5	33.33
Female	10	66.67
Age, years		
30-39	6	40
40-49	5	33.33
≥50	4	26.67
Education level		
Undergraduate	3	20
Master	7	46.67
Doctor	5	33.33
Professional title		
Intermediate	3	20
Associate senior	6	40
Senior	6	40
Field of work (multiplicity)		
Psychiatry and Mental Health	14	93.33
Neuropsychopharmacology	2	13.33
Applied psychology	1	6.67
Health management	1	6.67
Other	1	6.67
Professional experience, years		
1-10	3	20
11-20	5	33.33
21-30	3	20
31-40	4	26.67

#### 3.4.2 Reliability of expert consultation results

(1) Degree of activeness of experts: All questionnaires were completed and returned in both the first and second rounds, resulting in effective response rates of 100% for each round. In the first round, 80% of experts provided feedback, while 46.67% offered additional recommendations in the second round. These findings reflect a high level of engagement among the experts throughout the consultation process.

(2) Authority coefficient of experts: The Cs for two rounds of expert consultation were 0.89 in both rounds. The Ca was 0.91 and 0.96, respectively. Consequently, the Cr were 0.90 and 0.93, both exceeding the acceptable threshold of 0.7 (36). These results indicate that expert opinions from both consultations exhibit a high level of reliability.

(3) Degree of coordination and concentration of expert opinions: In the first round of consultation, the Cv ranged from 0.05 to 0.24, with item importance scores between 4.20 and 4.93, the full score frequency (K) from 40% to 93.33%, and a Kendall’s W between 0.129 and 0.267(*p* < 0.05). In the second round, the Cv ranged from 0.00 to 0.18, item importance scores were between 4.07 and 5.00, K ranged from 26.67% to 100%, and W varied from 0.149 to 0.492(*p* < 0.05), as detailed in **Table 6**, **7**.

**Table 6:** Degree of coordination and concentration of expert opinions in the two rounds.

Dimension/ Item no.	Round 1 (*n* = 15)				Round 2 (*n* = 15)			
	Item importance scores(*X ± SD*)	Cv	K(%)	Decision	Item importance scores(*X ± SD*)	Cv	K(%)	Decision
Functional literacy	4.93 ± 0.26	0.05	93.33	retained	5.00 ± 0.00	0.00	100	retained
Communicative literacy	4.80 ± 0.41	0.09	80	retained	5.00 ± 0.00	0.00	100	retained
Critical literacy	4.40 ± 0.83	0.19	60	retained	4.87 ± 0.35	0.07	86.67	retained
Numeracy	4.84 ± 0.35	0.10	73.33	retained	4.73 ± 0.46	0.10	73.33	retained
item1	4.80 ± 0.41	0.09	80	retained	4.93 ± 0.26	0.05	93.33	retained
item2	4.40 ± 0.51	0.12	40	retained	4.67 ± 0.49	0.10	66.67	retained
item3	4.87 ± 0.35	0.07	86.67	retained	4.87 ± 0.35	0.07	86.67	retained
item4	4.80 ± 0.56	0.12	86.67	retained	5.00 ± 0.00	0.00	100	retained
item5	4.47 ± 0.74	0.17	60	retained	4.93 ± 0.26	0.05	93.33	retained
item6	4.80 ± 0.56	0.12	86.67	retained	4.93 ± 0.26	0.05	93.33	retained
item7	4.53 ± 0.64	0.14	60	retained	4.93 ± 0.26	0.05	93.33	retained
item8	4.60 ± 0.63	0.14	66.67	retained	4.67 ± 0.49	0.10	66.67	retained
item9	4.40 ± 0.91	0.21	60	retained	4.93 ± 0.26	0.05	93.33	retained
item10	4.47 ± 0.74	0.17	60	retained	4.93 ± 0.26	0.05	93.33	retained
item11	4.33 ± 1.05	0.24	66.67	retained	4.07 ± 0.70	0.17	33.33	retained
item12	4.20 ± 0.94	0.22	46.67	retained	4.13 ± 0.74	0.18	33.33	retained
item13	4.93 ± 0.26	0.05	93.33	retained	4.53 ± 0.52	0.11	53.33	retained
item14	4.87 ± 0.35	0.07	86.67	retained	4.93 ± 0.26	0.05	93.33	retained
item15	4.27 ± 0.80	0.19	46.67	retained	4.93 ± 0.26	0.05	93.33	retained
item16	4.73 ± 0.46	0.10	73.33	retained	4.27 ± 0.70	0.16	40	retained
item17	4.67 ± 0.82	0.17	80	retained	5.00 ± 0.00	0.00	100	retained
item18	4.67 ± 0.62	0.13	73.33	retained	4.93 ± 0.26	0.05	93.33	retained
item19	4.80 ± 0.41	0.09	80	retained	4.93 ± 0.26	0.05	93.33	retained
item20	4.73 ± 0.46	0.10	73.33	retained	5.00 ± 0.00	0.00	100	retained
item21	4.40 ± 0.83	0.19	60	retained	5.00 ± 0.00	0.00	100	retained
item22	4.53 ± 0.64	0.14	60	retained	4.93 ± 0.26	0.05	93.33	retained
item23	4.27 ± 0.80	0.19	40	retained	4.93 ± 0.26	0.05	93.33	retained
item24	4.67 ± 0.62	0.13	73.33	retained	4.93 ± 0.26	0.05	93.33	retained
item25	4.60 ± 0.63	0.14	66.67	retained	4.93 ± 0.26	0.05	93.33	retained
item26	4.47 ± 0.83	0.19	60	retained	5.00 ± 0.00	0.00	100	retained
item27	4.20 ± 0.86	0.21	40	retained	4.27 ± 0.46	0.11	26.67	retained
item28	4.73 ± 0.46	0.10	73.33	retained	4.60 ± 0.51	0.11	60	retained
item29	4.73 ± 0.46	0.10	73.33	retained	4.73 ± 0.46	0.10	73.33	retained
item30	4.27 ± 0.80	0.19	46.67	retained	4.13 ± 0.74	0.18	33.33	retained
item31	4.47 ± 0.74	0.17	60	retained	4.93 ± 0.26	0.05	93.33	retained
item32	–	–	–	–	4.33 ± 0.49	0.11	33.33	retained
item33	–	–	–	–	4.33 ± 0.49	0.11	33.33	retained

Cv, coefficient of variation; K, the full score frequency; “-”, indicates that no relevant data are available.

**Table 7:** Coefficients of variation and expert coordination coefficients for indicators.

Indicator	Cv				W	χ^2^	p*-*Value
	Minimum	Maximum	Mean	SD			
First round							
Overall	0.05	0.24	0.138	0.050	0.164	83.818	<0.001
Functional literacy	0.05	0.21	0.121	0.045	0.165	22.313	0.008
Communication literacy	0.05	0.24	0.147	0.076	0.267	24.000	0.001
Critical literacy	0.09	0.19	0.146	0.039	0.129	21.251	0.031
Numeracy	0.10	0.21	0.141	0.050	0.188	14.073	0.015
Second round							
Overall	0.00	0.18	0.070	0.051	0.406	219.307	<0.001
Functional literacy	0.00	0.10	0.054	0.033	0.149	22.400	0.013
Communication literacy	0.00	0.18	0.105	0.071	0.492	44.271	<0.001
Critical literacy	0.00	0.11	0.041	0.034	0.432	71.235	<0.001
Numeracy	0.05	0.18	0.109	0.038	0.289	26.016	<0.001

W, Kendall’s Coefficient of Concordance; K, Full Score Frequency; SD, Standard Deviation.

First round: Functional literacy contains items 1-9, Communication literacy contains items 10-15, Critical literacy contains items 16-26, and Numeracy contains items 27-31.

Second round: Functional literacy contains items 1-10, Communication literacy contains items 11-16, Critical literacy contains items 17-27, and Numeracy contains items 28-33.

### 3.5 Final scale items

Following the first round of expert consultation, the dimensions were retained unchanged based on mean values and coefficient of variation (Cv). Two new items were added, 29 items were revised (12 item modifications and 17 response option adjustments), and scoring guidance was provided for one item. No items were deleted. In the second round, the dimensions were again retained, 2 additional items were added, and 20 items were revised (13 item modifications and 7 response option adjustments). Scoring guidance was also provided for one item, and no items were deleted. One proposed item (item 4), “Are you taking your medication on time and at the prescribed dosage?” was not accepted. This dichotomous format of this question would not align with the scale’s framework. Additionally, the phrasing risked social desirability bias, potentially compromising response accuracy. The original item, which uses specific options to assess the patient’s current medication regimen, was retained as it enhances understanding and supports individualized interventions. Details of the modifications from the two rounds of expert consultation were presented in **Supplementary Material 1**. The finalized Medication Literacy Scale for patients with mental disorders in recovery consists of four dimensions encompassing a total of 35 items: functional literacy (10 items), communicative literacy (6 items), critical literacy (11 items), and numeracy (8 items). The details have been shown in **Table 8**.

**Table 8:** The medication literacy assessment scale for patients with mental disorders in recovery.

The medication literacy assessment scale for patients with mental disorders in recovery		Score
**Functional Literacy**	1. You are taking your psychotropic medication as prescribed by your doctor □ Always □ Often □ Sometimes □ Rarely □ Never	□5 □4 □3 □2 □1
	2. Please name the psychotropic medication you are currently taking (refer to your pill box if needed).	□5 □4 □3 □2 □1
	3*. What effects does the psychotropic medication you are currently taking have? **Reference options:** □ Don’t know □ Antipsychotics □ Antidepressants □ Mood stabilizers □ Anxiolytics □ Sedative-hypnotics □ Cognitive enhancers	□5 □4 □3 □2 □1
	4. Please state your current medication status. **Reference options:** □ Don’t know □ On time and dosage □ On dosage and not on time □ On time and not on dosage □Neither on time nor on dosage □ Not taking medication	□5 □4 □3 □2 □1
	5*. Please specify the exact times you take your medication. **Reference options:** □ Don’t know □ Morning fasting □ Taking before meal □ Taking during meal □ Taking after meal □ Taking before bedtime	□5 □4 □3 □2 □1
	6. Please specify the total duration for which you should continue taking your current psychiatric medication. **Reference options:** □ Don’t know □ Continuous medication, 1-3 years for first episode □ Continuous medication, 3-5 years for single relapse □ Long-term medication for more than two relapses within 5 years □ Long-term treatment for significant residual symptoms □ As directed by doctor	□5 □4 □3 □2 □1
	7. Please describe your follow-up appointments after taking psychotropic medication. **Reference options:** □ Don’t know □ No need for follow-up □ Strictly adherence to doctor’s orders □ Early or delayed follow-up according to your condition □ Follow-up visit based on personal preference □ Follow-up by your family members	□5 □4 □3 □2 □1
	8*. You are required to undergo routine examinations related to your psychotropic medication during treatment. **Reference options:** □ Don’t know □ Laboratory tests (blood counts, liver and kidney functions, etc.) □ Electrocardiogram □ B-scan ultrasonography □ Electroencephalography □ Neuropsychological evaluation	□5 □4 □3 □2 □1
	9*. Please list the common side effects of the psychotropic medication you are currently taking. **Reference options:** □ Don’t know □ General common adverse reactions (gastrointestinal symptoms, cardiovascular system symptoms) □ extra vertebral system reactions (acute dystonia, Parkinson’s-like disease, inability to sit still, and delayed dyskinesia) □ Metabolic syndrome (weight gain, hyperglycemia, hyperlipidemia, and hypertension) □ Disorders of the endocrine system (increased prolactin, menstrual disorders, and abnormalities of sexual function) □ Abnormalities of liver and kidney function □ Excessive Sedation, insomnia, irritability □ Lithium toxicity □ Leukopenia □ Other, please list:	□5 □4 □3 □2 □1
	10*. Please specify the precautions you should take while using psychotropic medication. **Reference options:** □ Don’t know □ Monitor medication dosage □ Check medication expiration date □ Understand drug interactions □ Be aware of adverse drug reactions □ Know indications and contraindications □ Knowing the special medication population (pregnant women, children, elderly, etc.) □ Other, please list:	□5 □4 □3 □2 □1
**Communicative Literacy**	11. How often do you access information on psychotropic medication from public sources, like books, internet? □ Always □ Often □ Sometimes □ Rarely □ Never	□5 □4 □3 □2 □1
	12. How often do you access information on psychotropic medication from a loved one or friend? □ Always □ Often □ Sometimes □ Rarely □ Never	□5 □4 □3 □2 □1
	13. How often do you access information on psychotropic medication from health education lectures held by hospitals or the community? □ Always □ Often □ Sometimes □ Rarely □ Never	□5 □4 □3 □2 □1
	14. How often do you consult healthcare professionals about information related to your current psychotropic medication (such as side effect precautions, risks and benefits, or adjustment methods)? □ Always □ Often □ Sometimes □ Rarely □ Never	□5 □4 □3 □2 □1
	15. How often do you report any adverse reactions or side effects from your current psychotropic medication to healthcare professionals? □ Always □ Often □ Sometimes □ Rarely □ Never	□5 □4 □3 □2 □1
	16. How often do you participate in peer support groups or group interventions to discuss your current psychotropic medication? □ Always □ Often □ Sometimes □ Rarely □ Never	□5 □4 □3 □2 □1
**Critical Literacy**	17. Please indicate the typical time it takes for your current psychotropic medication to take effect after administration. **Reference options:** □ Don’t know □ Within 24 hours □ Within 1 week □ 2-3 weeks □ After one month	□5 □4 □3 □2 □1
	18*. Please indicate the factors that may affect medication effectiveness during treatment. **Reference options:** □ Don’t know □ Switched to a different manufacturer for the same medication □ Changed medication type □ Changed medication timing □ Adjusted medication dosage □ Adjusted medication frequency □ Stopped taking medication □ Irregular lifestyle or routine □ Smoking, alcohol, strong tea, or coffee, etc. □ Other, please list:	□5 □4 □3 □2 □1
	19. What should you do if you miss a dose of your medication? **Reference options:** □ Don’t know □ Ignore □ Take double dose with next dose □ Skip the missed dose and take the next dose as scheduled □ Time between next dose of medication □ Consult psychiatrists □ Other, please list:	□5 □4 □3 □2 □1
	20. What actions do you usually take if you accidentally take the wrong dose or an overdose of your medication? **Reference options:** □ Don’t know □ Ignore □ Attempt to accelerate metabolism (e.g., self-induced vomiting) □ Consult psychiatrists immediately □ Other, please list:	□5 □4 □3 □2 □1
	21. How do you usually manage changes in your condition while on medication? **Reference options:** □ Don’t know □ Self-discontinuation of medication □ Adhere to the prescribed medication dosage and review at the same time □ Self-adjustment of dosage of medication □ Consult psychiatrists immediately □ Other, please list:	□5 □4 □3 □2 □1
	22. What do you usually do if you feel that your medication is ineffective? **Reference options:** □ Don’t know □ Self-discontinuation of medication □ Adhere to the prescribed medication dosage and review at the same time □ Self-adjustment of dosage of medication □Consult psychiatrists immediately □ Other, please list:	□5 □4 □3 □2 □1
	23*. How do you manage side effects while taking your medication? **Reference options:** □ Don’t know □ Actively manage, and handling common or persistent side effects according to psychiatrist’s guidance (e.g., using laxatives for constipation) □ Passively cope, such as adjusting the dose or stopping the medication on your own □ Ignore unavoidable medication side effects □ Consult psychiatrists immediately for severe side effects □ Constant side effects, no special attention needed □ Take no action □ Other, please list:	□5 □4 □3 □2 □1
	24*. Please state the indications for discontinuing your current psychotropic medication. **Reference options:** □ Don’t know □ Discontinue when symptoms disappear □ Discontinue if perceived as ineffective □ Discontinue as per doctor’s instructions □ Lifelong medication, cannot discontinue □ For first episode, taper off medication after the maintenance phase □ For recurrent cases, taper off if the condition remains stable for over 3 years with no significant fluctuations □ Discontinue if serious drug-related adverse effects occur (e.g., malignant syndrome, myocarditis, agranulocytosis) □ Other, please list:	□5 □4 □3 □2 □1
	25*. Please describe the potential risks of interrupting your medication during treatment. **Reference options:** □ Don’t know □ No impact □ Withdrawal syndrome (dizziness, pain, inexplicable discomfort, anxiety, tachycardia, etc.) □ Relapse or worsening of mental illness □ Affecting the recovery effect □ Increasing the cost of treatment	□5 □4 □3 □2 □1
	26. How do you typically handle situations when your prescribed treatment conflicts with your personal preferences for taking medication? **Reference options:** □ Don’t know □ Take medication according to personal preference □ Follow the prescribed medication □ Actively discuss medication options with the doctor □ Other, please list:	□5 □4 □3 □2 □1
	27. What do you consider to be the most critical points in assessing the efficacy of psychotropic medications? **Reference options:** □ Don’t know □ Psychotic symptoms □ Physical condition □ Social functioning □ Daily living abilities □ Insight recovery □ Other, please list:	□5 □4 □3 □2 □1
**Numeracy**	28. Please converting gram (g) to milligrams (mg) on the medication packaging (e.g., 1g =? mg; 0.1g =? mg). □ Fully aware □ Knows enough □ Knows some □ Knows a little □ Doesn’t know at all	□5 □4 □3 □2 □1
	29. Please calculate the number of tablets required for the prescribed dosage each time. □ Fully aware □ Knows enough □ Knows some □ Knows a little □ Doesn’t know at all	□5 □4 □3 □2 □1
	30. Please specify the number of times per day you take your current psychotropic medication. □ Fully aware □ Knows enough □ Knows some □ Knows a little □ Doesn’t know at all	□5 □4 □3 □2 □1
	31. Please specify the maximum daily dosage of your current medication. □ Fully aware □ Knows enough □ Knows some □ Knows a little □ Doesn’t know at all	□5 □4 □3 □2 □1
	32. Please specify the toxic dose of your current medication. □ Fully aware □ Knows enough □ Knows some □ Knows a little □ Doesn’t know at all	□5 □4 □3 □2 □1
	33. Please calculate how long your remaining psychotropic medication will last. □ Fully aware □ Knows enough □ Knows some □ Knows a little □ Doesn’t know at all	□5 □4 □3 □2 □1
	34. Please state the expiration date of your current psychotropic medication. □ Fully aware □ Knows enough □ Knows some □ Knows a little □ Doesn’t know at all	□5 □4 □3 □2 □1
	35. Please state the date of your next follow-up appointment as agreed upon with your doctor. □ Fully aware □ Knows enough □ Knows some □ Knows a little □ Doesn’t know at all	□5 □4 □3 □2 □1
	**Totals:**	

*, Indicates multiple-choice questions. Each question includes detailed scoring criteria, which can be converted to a five-point Likert scale. Scoring details supporting this scale are available from the corresponding author upon reasonable request. Reference options: In this question, you can select one or more answers based on the actual situation. Totals: Represents the sum or total of the scores of all items.

## 4 Discussion

### 4.1 Summary of the findings

Medication literacy is essential for patients with mental disorders as it directly influences their ability to manage symptoms and prevent relapses. However, the absence of effective assessment tools has limited understanding of patients’ medication-related knowledge and behaviors. This study addresses this gap by developing and validating a Medication Literacy Assessment Scale for patients with mental disorders in recovery through a rigorous Delphi process, integrating insights from a comprehensive literature analysis and qualitative interview findings. Expert consensus confirmed the scale’s relevance and comprehensiveness, underscoring its potential as a systematic approach to evaluating patients’ understanding and application of psychotropic medications.

### 4.2 Comprehensiveness of the medication literacy assessment scale

This study is grounded in the conceptual model of medication literacy (5), which serves as a robust theoretical framework for the development of the Medication Literacy Assessment Scale. The model identifies the knowledge and skills that individuals need to effectively use medication information and achieve medication literacy goals. By incorporating these broader principles, the model provided clear guidance for constructing a scale that is both comprehensive and tailored to the specific challenges faced by patients with mental disorders in recovery.

The comprehensiveness of the Medication Literacy Assessment Scale for patients with mental disorders in recovery was supported by integrating findings from an extensive literature review with qualitative input from real-world clinical experiences. The literature review incorporated existing medication literacy and knowledge assessment tools (38, 39, 45, 46) and authoritative guidelines for assessing and managing mental disorders (28, 29), ensuring that the scale captured a broad spectrum of knowledge and skills essential for medication literacy in mental disorders. Semi-structured interviews with psychiatric professionals and patients provided additional clinical insights, highlighting practical challenges encountered in medication management and identifying core competencies critical to effective care. Building on previous instruments that primarily focused on patient attitudes toward side effects (47, 48), the scale developed in this study adopts a more comprehensive approach by also assessing behavioral responses and coping strategies essential for self-management. Different from instruments that assess only single aspects such as mental illness knowledge and medication attitudes (19, 47, 49), this study fills the gap in the systematic exploration of medication literacy in patients with mental disorders. It allows for dynamic and multidimensional assessment by including items that reflect patients’ practical understanding and decision-making related to medication use. For example, items such as “your follow-up appointments after taking psychotropic medication”, “What actions do you usually take if you accidentally take the wrong dose or an overdose of your medication”, and “the potential risks of interrupting your medication during treatment”, etc. Furthermore, the scale introduces dimensions that are often overlooked in existing tools, including critical literacy dimension, such as “the indications for discontinuing your current psychotropic medication”, and the calculation ability dimension, as reflected in item like “the date of your next follow-up appointment as agreed upon with your doctor”. These additions further enhance the clinical relevance and depth of the assessment. Additionally, different from earlier tools with a limited medication scope, this scale covers a wide range of commonly prescribed psychotropic drugs, including antipsychotics, antidepressants, mood stabilizers, anxiolytics, and sedatives, etc. (50). It is aligned with the WHO’s ‘5 Moments for Medication Safety’ framework (Starting medication, Taking medication, Adding medication, Reviewing medications, and Stopping medication) (1), enabling structured assessment across the full course of treatment. Overall, these features contribute to the scale’s practical utility in psychiatric care. By providing a more comprehensive understanding of patients’ medication literacy, this scale could inform tailored intervention, such as simplified medication instructions, educational counseling, or closer monitoring to enhance adherence and promote safer, more effective medication use during recovery.

### 4.3 Scientific validity and reliability of the medication literacy assessment scale

The validity and reliability of the scale were further strengthened through Delphi process. The expert panel in this study, consisting of professionals with advanced qualifications (80% holding a master’s degree or higher) and extensive experience in psychiatry, provided a solid foundation for item refinement. The 100% response rates across two rounds of consultation reflect both the rationality of the research method and the high level of engagement from the experts, ensuring a thorough review process. The authority factors, exceeding 0.9 in both rounds, indicate a high level of familiarity with the content, allowing for a well-reasoned and authoritative basis for feedback. The Kendall coefficients of 0.164 and 0.406 suggest that expert opinions became more aligned through the rounds, particularly in the second round, where high coordination coefficients confirmed the reliability and consistency of the consensus achieved.

### 4.4 Clinical implication of the medication literacy assessment scale

The Medication Literacy Assessment Scale provides a structured approach that may enhance medication literacy among patients with mental disorders in recovery, supporting recovery-oriented care through targeted interventions and systematic evaluation. By identifying literacy gaps in medication management, this scale could allow psychiatric professionals to tailor educational efforts to address specific adherence challenges and strengthen patient engagement. Its adaptability across various settings, including inpatient, outpatient, and community programs, could support continuity of care and might allow integration into electronic health records, facilitating more seamless access to literacy assessments for the entire care team. Additionally, insights from the scale may also guide caregiver involvement, which can be especially beneficial for patients requiring additional support. Furthermore, regular reassessment with this scale could enable psychiatric professionals to monitor literacy progress over time, while integration with digital health tools might broaden access to self-management resources, potentially empowering patients to engage more effectively with their medication regimen.

### 4.5 Strengths and limitations

This study initiates the development of a Medication Literacy Assessment Scale tailored for patients with mental disorders in recovery. Drawing on the current medication-taking practices of this group, the study explores medication literacy across ‘functional literacy,’ ‘communicative literacy,’ ‘critical literacy,’ and ‘numeracy’ dimensions. Through semi-structured interviews and expert feedback, this study establishes evaluation items that can serve as a quantitative tool for assessing medication literacy in these patients. Additionally, this study applied elements of the COSMIN framework to guide the early stages of scale development, prioritizing clarity and minimizing potential design-stage bias. Although further statistical validation, including exploratory and confirmatory factor analyses, is needed to rigorously confirm the scale’s structure, these initial steps provide a foundation for a transparent and systematic development process.

Several limitations must be acknowledged in this study. The item pool was developed based on a literature review, rather than a systematic review, due to the limited number of studies on medication literacy in psychiatric populations. Although this approach facilitated the use of available evidence, it may have introduced bias and limited coverage. Additionally, the specific challenges that patients with mental disorders may face in understanding medication information could limit the scale’s comprehensiveness and its ability to fully capture all relevant facets of medication literacy. The expert panel, comprising only 15 members from China, may limit the cross-cultural applicability and generalizability of the findings. The Delphi method, while useful, depends on subjective expert judgments, introducing potential bias as individual backgrounds and perspectives could influence the selection of items and structure of the scale. Although email-based consultations-maintained anonymity, the absence of direct interaction might have limited the depth of discussion and precision in refining item selection. while preliminary findings suggest the scale is promising, further assessment of its psychometric properties and statistical validation of its factor structure are needed to confirm its robustness and support broader applicability across different populations. Additionally, future research should focus on the scale’s clinical application, including refinement of its structure and the establishment of cutoff scores to support risk stratification. This will facilitate standardized interpretation and practical use in real-world settings. Further studies may also examine the use of Medication Literacy Assessment Scale results to inform personalized interventions to enhance medication adherence based on patients’ literacy levels. Notably, individuals with intellectual disability, cognitive impairments, or high-risk psychiatric symptoms were excluded to ensure response validity. However, this may limit the Medication Literacy Assessment Scale generalizability to vulnerable populations. Future research should explore adapted or proxy-assisted versions for more inclusive assessment.

## 5 Conclusions

This Delphi study provided valuable insights that supported the development of the Medication Literacy Assessment Scale. It offers a scientifically grounded tool that may assist psychiatric healthcare providers in the quantitative assessment of medication literacy and in identifying high-risk populations. While the tool shows promise, its psychometric properties remain untested. Further assessment of its reliability, validity, and structure, as well as cross-cultural piloting, are needed before it can be used in practice.

## Data Availability

The raw data supporting the conclusions of this article will be made available by the authors, without undue reservation.

## References

[1] Organization WH. Medication without harm: policy brief. World Health Organization (2024). Available online at: https://www.who.int/publications/i/item/9789240062764.

[2] England NHS and NHS Improvement. The NHS Patient Safety Strategy: Safer culture, safer systems, safer patients London NHS. (2019). Available online at: https://www.england.nhs.uk/patient-safety/the-nhs-patient-safety-strategy/ (Accessed November 25, 2024).

[3] China NHCotPsRo. A circular on further strengthening medication safety management and enhancing the level of rational medication use. (2022) . Available online at: https://www.gov.cn/zhengce/zhengceku/2022-07/30/content_5703604.htm (Accessed November 4, 2024).

[4] Pouliot A Vaillancourt R Stacey D Suter P. Defining and identifying concepts of medication literacy: An international perspective. Res Soc Adm Pharm. (2018) 14:797–804. doi: 10.1016/j.sapharm.2017.11.005 29191647

[5] Neiva Pantuzza LL Nascimento ED Crepalde-Ribeiro K Botelho SF Parreiras Martins MA Camila de Souza Groia Veloso R. Medication literacy: A conceptual model. Res Soc Adm Pharm. (2022) 18:2675–82. doi: 10.1016/j.sapharm.2021.06.003 34134939

[6] Ni H Lin Y Peng Y Li S Huang X Chen L. Relationship between family functioning and medication adherence in chinese patients with mechanical heart valve replacement: A moderated mediation model. Front Pharmacol. (2022) 13:817406. doi: 10.3389/fphar.2022.817406 35273498 PMC8902640

[7] Zaeh SE Ramsey R Bender B Hommel K Mosnaim G Rand C. The impact of adherence and health literacy on difficult-to-control asthma. J Allergy Clin Immunol Pract. (2022) 10:386–94. doi: 10.1016/j.jaip.2021.11.003 PMC10207170 34788658

[8] Marcum ZA Hanlon JT Murray MD. Improving medication adherence and health outcomes in older adults: an evidence-based review of randomized controlled trials. Drugs Aging. (2017) 34:191–201. doi: 10.1007/s40266-016-0433-7 28074410 PMC5325769

[9] Lappalainen M Härkänen M Laukkanen E Kuosmanen L. Effectiveness of providing information on antipsychotic medication to patients with psychotic disorders: an integrative review. Issues Ment Health Nurs. (2023) 44:373–86. doi: 10.1080/01612840.2023.2195507 37126802

[10] Lau KC Lee EH Hui CL Chang WC Chan SK Chen EY. Psychosis patients’ knowledge, adherence and attitudes towards the naming of antipsychotic medication in Hong Kong. Early Interv Psychiatry. (2015) 9:422–7. doi: 10.1111/eip.2015.9.issue-5 25244594

[11] Al Hathloul AM Al Jafer MA Al Fraih IA. Psychiatric patients awareness of their illnesses and medications. Neurosci (Riyadh). (2016) 21:37–42. doi: 10.17712/nsj.2016.1.20150481 PMC5224409 26818165

[12] Bhattacharyya S. Association of medication nonadherence with increased risk of violence to others among patients with schizophrenia. JAMA Netw Open. (2023) 6:e235784. doi: 10.1001/jamanetworkopen.2023.5784 37017972

[13] Tan W Lin H Lei B Ou A He Z Yang N. The psychosis analysis in real-world on a cohort of large-scale patients with schizophrenia. BMC Med Inform Decis Mak. (2020) 20:132. doi: 10.1186/s12911-020-1125-0 32646484 PMC7477870

[14] Semahegn A Torpey K Manu A Assefa N Tesfaye G Ankomah A. Psychotropic medication non-adherence and its associated factors among patients with major psychiatric disorders: a systematic review and meta-analysis. Syst Rev. (2020) 9:17. doi: 10.1186/s13643-020-1274-3 31948489 PMC6966860

[15] Deng M Zhai S Ouyang X Liu Z Ross B. Factors influencing medication adherence among patients with severe mental disorders from the perspective of mental health professionals. BMC Psychiatry. (2022) 22:22. doi: 10.1186/s12888-021-03681-6 34996394 PMC8740063

[16] Chai X Liu Y Mao Z Li S. Barriers to medication adherence for rural patients with mental disorders in eastern China: a qualitative study. BMC Psychiatry. (2021) 21:141. doi: 10.1186/s12888-021-03144-y 33685432 PMC7941940

[17] Erdmann T Berwian IM Stephan KE Seifritz E Walter H Huys QJM. Amygdala reactivity, antidepressant discontinuation, and relapse. JAMA Psychiatry. (2024) 81(11):1081–9. doi: 10.1001/jamapsychiatry.2024.2136 PMC11391364 39259548

[18] Yeisen RAH Bjornestad J Joa I Johannessen JO Opjordsmoen S. Experiences of antipsychotic use in patients with early psychosis: a two-year follow-up study. BMC Psychiatry. (2017) 17:299. doi: 10.1186/s12888-017-1425-9 28830453 PMC5567881

[19] Nordon C Falissard B Gerard S Angst J Azorin JM Luquiens A. Patient satisfaction with psychotropic drugs: Validation of the PAtient SAtisfaction with Psychotropic (PASAP) scale in patients with bipolar disorder. Eur Psychiatry. (2014) 29:183–90. doi: 10.1016/j.eurpsy.2013.03.001 23769326

[20] Ascher-Svanum H. Development and validation of a measure of patients’ knowledge about schizophrenia. Psychiatr Serv. (1999) 50:561–3. doi: 10.1176/ps.50.4.561 10211743

[21] Gulliver A Griffiths KM Christensen H Mackinnon A Calear AL Parsons A. Internet-based interventions to promote mental health help-seeking in elite athletes: an exploratory randomized controlled trial. J Med Internet Res. (2012) 14:e69. doi: 10.2196/jmir.1864 22743352 PMC3414855

[22] Kiropoulos LA Griffiths KM Blashki G. Effects of a multilingual information website intervention on the levels of depression literacy and depression-related stigma in Greek-born and Italian-born immigrants living in Australia: a randomized controlled trial. J Med Internet Res. (2011) 13:e34. doi: 10.2196/jmir.1527 21504872 PMC3221382

[23] Sauceda JA Loya AM Sias JJ Taylor T Wiebe JS Rivera JO. Medication literacy in Spanish and English: psychometric evaluation of a new assessment tool. J Am Pharm Assoc (2003). (2012) 52:e231–40. doi: 10.1331/JAPhA.2012.11264 23229985

[24] Yeh YC Lin HW Chang EH Huang YM Chen YC Wang CY. Development and validation of a Chinese medication literacy measure. Health Expect. (2017) 20:1296–301. doi: 10.1111/hex.2017.20.issue-6 PMC5689244 28474423

[25] Horvat N Kos M. Development, validation and performance of a newly designed tool to evaluate functional medication literacy in Slovenia. Int J Clin Pharm. (2020) 42:1490–8. doi: 10.1007/s11096-020-01138-6 32885323

[26] Terwee CB Prinsen CAC Chiarotto A Westerman MJ Patrick DL Alonso J. COSMIN methodology for evaluating the content validity of patient-reported outcome measures: a Delphi study. Qual Life Res. (2018) 27:1159–70. doi: 10.1007/s11136-018-1829-0 PMC5891557 29550964

[27] Barnes TR Drake R Paton C Cooper SJ Deakin B Ferrier IN. Evidence-based guidelines for the pharmacological treatment of schizophrenia: Updated recommendations from the British Association for Psychopharmacology. J Psychopharmacol. (2020) 34:3–78. doi: 10.1177/0269881119889296 31829775

[28] National Institute for Health and Care Excellence: Guidelines. Bipolar disorder: assessment and management. London: National Institute for Health and Care Excellence (NICE) Copyright © NICE 2024 (2023). 31556980

[29] National Institute for Health and Care Excellence: Guidelines. Depression in adults: treatment and management. London: National Institute for Health and Care Excellence (NICE) Copyright © NICE 2022 (2022). 35977056

[30] Graneheim UH Lundman B. Qualitative content analysis in nursing research: concepts, procedures and measures to achieve trustworthiness. Nurse Educ Today. (2004) 24:105–12. doi: 10.1016/j.nedt.2003.10.001 14769454

[31] Jünger S Payne SA Brine J Radbruch L Brearley SG. Guidance on Conducting and REporting DElphi Studies (CREDES) in palliative care: Recommendations based on a methodological systematic review. Palliat Med. (2017) 31:684–706. doi: 10.1177/0269216317690685 28190381

[32] Hasson F Keeney S McKenna H. Research guidelines for the Delphi survey technique. J Adv Nurs. (2000) 32:1008–15. doi: 10.1046/j.1365-2648.2000.t01-1-01567.x 11095242

[33] Preston CC Colman AM. Optimal number of response categories in rating scales: reliability, validity, discriminating power, and respondent preferences. Acta Psychol (Amst). (2000) 104:1–15. doi: 10.1016/S0001-6918(99)00050-5 10769936

[34] Dawes J. Do data characteristics change according to the number of scale points used? An experiment using 5-point, 7-point and 10-point scales. Int J Market Res. (2008) 50:61–104. doi: 10.1177/147078530805000106

[35] Xu H Dong C Yang Y Sun H. Developing a professional competence framework for the master of nursing specialist degree program in China: A modified Delphi study. Nurse Educ Today. (2022) 118:105524. doi: 10.1016/j.nedt.2022.105524 36084450

[36] Zamperoni G Tan EJ Sumner PJ Rossell SL. Exploring the conceptualisation, measurement, clinical utility and treatment of formal thought disorder in psychosis: A Delphi study. Schizophr Res. (2024) 270:486–93. doi: 10.1016/j.schres.2024.06.042 39002286

[37] Liu Y Li X Yang M Ding Y Ji M. Screening indicators to evaluate the clinical significance of drug-drug interactions in polypharmacy among older adults with psychiatric disorders: a delphi study. BMC Psychiatry. (2024) 24:417. doi: 10.1186/s12888-024-05872-3 38834965 PMC11151475

[38] Maniaci MJ Heckman MG Dawson NL. Functional health literacy and understanding of medications at discharge. Mayo Clin Proc. (2008) 83:554–8. doi: 10.1016/S0025-6196(11)60728-3 18452685

[39] Stilley CS Terhorst L Flynn WB Fiore RM Stimer ED. Medication health literacy measure: development and psychometric properties. J Nurs Meas. (2014) 22:213–22. doi: 10.1891/1061-3749.22.2.213 PMC4580338 25255674

[40] Zhou H. Study on development and test of the Medication Literacy Scale for Caregivers of Patients with Schizophrenia. (Master's thesis). Kunming Medical University (2022). doi: 10.27202/d.cnki.gkmyc.2022.001088

[41] Zhang N Wang L Ouyang YQ Redding S. Survey on medication information literacy and influencing factors among pregnant Chinese women. J Matern Fetal Neonatal Med. (2021) 34:1619–26. doi: 10.1080/14767058.2019.1642869 31331258

[42] Jang SM Parker WM Pai AB Jiang R Cardone KE. Assessment of literacy and numeracy skills related to medication labels in patients on chronic in-center hemodialysis. J Am Pharm Assoc (2003). (2020) 60:957–62.e1. doi: 10.1016/j.japh.2020.07.010 32811751

[43] Zhong Z Shi S Duan Y Shen Z Zheng F Ding S. The development and psychometric assessment of chinese medication literacy scale for hypertensive patients (C-MLSHP). Front Pharmacol. (2020) 11:490. doi: 10.3389/fphar.2020.00490 32425773 PMC7203424

[44] Lee CH Chang FC Hsu SD Chi HY Huang LJ Yeh MK. Inappropriate self-medication among adolescents and its association with lower medication literacy and substance use. PloS One. (2017) 12:e0189199. doi: 10.1371/journal.pone.0189199 29240799 PMC5730183

[45] Pantuzza LLN do Nascimento E Botelho SF da Rocha ALP Martins MAP do Nascimento MMG. Development and content validation of the medication literacy test for older adults (TELUMI). Arch Gerontol Geriatr. (2023) 112:105027. doi: 10.1016/j.archger.2023.105027 37080136

[46] Evans-Lacko S Little K Meltzer H Rose D Rhydderch D Henderson C. Development and psychometric properties of the Mental Health Knowledge Schedule. Can J Psychiatry. (2010) 55:440–8. doi: 10.1177/070674371005500707 20704771

[47] Wykes T Evans J Paton C Barnes TRE Taylor D Bentall R. What side effects are problematic for patients prescribed antipsychotic medication? The Maudsley Side Effects (MSE) measure for antipsychotic medication. Psychol Med. (2017) 47:2369–78. doi: 10.1017/S0033291717000903 PMC5820531 28420450

[48] Hynes C Keating D McWilliams S Madigan K Kinsella A Maidment I. Glasgow Antipsychotic Side-effects Scale for Clozapine - Development and validation of a clozapine-specific side-effects scale. Schizophr Res. (2015) 168:505–13. doi: 10.1016/j.schres.2015.07.052 26276305

[49] Wang M Zhao M Zhang W Li W He R Ding R. Knowledge about schizophrenia test: the Chinese Mandarin version and its sociodemographic and clinical factors. BMC Psychiatry. (2023) 23:535. doi: 10.1186/s12888-023-04822-9 37488539 PMC10367326

[50] Martins MJRV Pinto AM Castilho P Macedo AF Pereira AT Bajouco M. Assessing beliefs and attitudes towards antipsychotic medication from a recovery-based perspective: Psychometric properties of a new scale. Psychiatry Res. (2019) 273:325–30. doi: 10.1016/j.psychres.2019.01.043 30677722

