# Supplementary material for: Development of a medication literacy assessment scale for patients with mental disorders in recovery in China: a mixed-methods Delphi study
Source: Front Psychiatry. 2025 May 27;16:1551160. doi: 10.3389/fpsyt.2025.1551160 (PMC12148901; doi:10.3389/fpsyt.2025.1551160)
Supplement: Supplementary file 1 [file Table1.docx]

**Development of** **a Medication Literacy Assessment Scale for Patients with Mental Disorders in Recovery in China: A Delphi Study**

**Supplementary etable 1 1**

**Supplementary etable 2 4**

**Supplementary etable 3 9**

**Supplementary etable 1 The initial item pool of medication literacy for patients with mental disorders in recovery**

| **Dimension** | **Item** | **Origin** |
| --- | --- | --- |
| **Functional Literacy** | 1. Ability to read and write independently. (Boxes for psychotropic medication are given for testing or judgment based on years of schooling). | Literature |
|  | 2. Please name the psychiatric medication you are currently taking. | Literature |
|  | 3. Know the types of psychiatric medication (antipsychotics, antidepressants, mood stabilizers, etc.). | Literature, interviews |
|  | 4. Whether the purpose of current psychiatric medication is known. | Literature,guidelines and consensus,interviews |
|  | 5. Are you aware of the expiration date of the psychiatric medication you are currently taking? | Literature |
|  | 6. Time to take medication (before, during, and after meals). | Literature, interviews |
|  | 7. Evaluate if the psychiatric medication taken interacts with other treatments, such as hypoglycemic, antihypertensive, or antipyretic medications. | Literature, interviews |
|  | 8. Knowledge of indications for taking psychotropic medication. | Literature |
|  | 9. Knowledge of contraindications for taking psychotropic medication. | Literature |
|  | 10. Side effects of psychotropic medication currently taken (extra vertebral reactions, Parkinson's syndrome, etc.). | Literature, guidelines and consensus,interviews |
|  | 11. Interactions between current psychiatric medications and alcohol. | Literature, interviews |
|  | 12. Interactions between current psychiatric medications and food. | Literature,interviews |
|  | 13. Indications for discontinuation of psychotropic medication. | Literature,guidelines and consensus,interviews |
|  | 14. How long does it take for psychotropic medication to start working? | guidelines and consensus,interviews |
|  | 15. How to store psychiatric medication. | Literature, interviews |
|  | 16. Understand the medication disposition instructions, including the pharmacokinetics involved. | Literature |
|  | 17. whether it is feasible to comprehend the information delineated in the instructions. | Literature,interviews |
| **CommunicativeLiteracy** | 18. Access information about psychotropic medication from written sources, such as prescriptions, instructions, newspapers, and other materials. | Literature, interviews |
|  | 19. Access information about psychotropic medication from verbal sources, such as healthcare professionals, relatives, friends, peers, etc. | Literature, interviews |
|  | 20. Access information about psychotropic medication online, such as smartphones, computers, and other electronic devices. | Literature,interviews |
|  | 21. Access information about psychotropic medications by attending online or offline health education seminars. | Literature,interviews |
|  | 22. What are some additional methods to obtain more information about psychotropic medication? | Literature,interviews |
|  | 23. Communicating effectively with healthcare providers using listening, speaking, reading, writing, and body language. | Literature, interviews |
|  | 24. Regularly communicate with healthcare professionals or patients about their current feelings regarding the use of psychotropic medication. | Literature, guidelines and consensus,interviews |
|  | 25. Engagement in peer support groups. | Literature,guidelines and consensus |
| **Critical**  **Literacy** | 26. Whether information about psychotropic medication comes from official sources. | Literature, interviews |
|  | 27. Ability to recognize medication advertisements. | Literature |
|  | 28. Ability to select the required medication information from a large amount of information. | Literature |
|  | 29. Are the labeling instructions on the boxes provided by the pharmacy being followed? | Literature, interviews |
|  | 30. Understanding and acting on specific information about psychotropic medication, such as lithium dosage. | Literature,interviews |
|  | 31. The ability to make informed and accurate health medication decisions. | Literature, interviews |
|  | 32. When to seek help, particularly in an overdose. | Guidelines and consensus,interviews |
|  | 33. When scheduling a follow-up appointment or dispensing your medication. | guidelines and consensus,interviews |
|  | 34. Ability to make an appointment with an outpatient doctor. | guidelines and consensus,interviews |
|  | 35. Whether autonomously modifying the dosage of psychotropic medication. | guidelines and consensus,interviews |
|  | 36. Whether autonomously discontinuing the dosage of psychotropic medication. | guidelines and consensus,interviews |
|  | 37. How to deal with psychotropic medication when it is missed or forgotten? | guidelines and consensus,interviews |
|  | 38. How to deal with psychotropic medication when it is misused, overused, or underused? | guidelines and consensus,interviews |
|  | 39. How to deal with psychotropic medication doesn't work when self-perceived? | guidelines and consensus,interviews |
|  | 40. Individuals should be able to observe their reactions to medication and compare them with the adverse reactions listed in the drug inserts. | Literature, interviews |
|  | 41. The ability to distinguish between psychotropic medication’s common and specific side effects. | Literature, interviews |
|  | 42. How do you deal with specific side effects like muscle tension, stiffness, or instability while walking? | Literature, interviews |
| **Numeracy** | 43. The capability to perform addition, subtraction, multiplication, and division operations. | Literature |
|  | 44. Is it possible to distinguish between grams (g) and milligrams (mg) on the pillbox? | Literature |
|  | 45. Calculate the dose of each medication for every meal. | Literature, interviews |
|  | 46. Calculate how many times each medication should be taken each day. | Literature, interviews |
|  | 47. The intervals for medication administration are calculated with precision. | Literature, interviews |
|  | 48. What is the maximum daily or session dose of psychotropic medication? | Literature, interviews |
|  | 49. Calculate the date and time for your next follow-up visit to the clinic. | Literature, interviews |

**Supplementary etable 2 The initial expert-reviewed version of the Medication Literacy Assessment Scale**

**E table 2.1 The dimension of the Medication Literacy Assessment Scale**

| **Dimensions** | **Expert Opinion** | | | | | |
| --- | --- | --- | --- | --- | --- | --- |
|  | **Importance Score** | | | | | **Revised Opinion** |
|  | 5 | 4 | 3 | 2 | 1 |  |
| **Functional Literacy** | □ | □ | □ | □ | □ |  |
| **Communication Literacy** | □ | □ | □ | □ | □ |  |
| **Critical Literacy** | □ | □ | □ | □ | □ |  |
| **Numeracy** | □ | □ | □ | □ | □ |  |
| **Please identify the items that need to be added and provide clear reasons for your suggestions.** |  | | | | | |

**E table 2.2 The items of the Medication Literacy Assessment Scale**

| **Items** | | **Expert Opinion** | | | | | |
| --- | --- | --- | --- | --- | --- | --- | --- |
|  |  | **Importance Score** | | | | | **Revised Opinion** |
|  |  | **5** | **4** | **3** | **2** | **1** |  |
| 1. Please name the psychotropic medication you are currently taking (refer to your pill box if needed). | | □ | □ | □ | □ | □ |  |
| 2*. Please answer the type of psychotropic medication you are currently taking. **Reference options:** □ Don’t know □Antipsychotics □Antidepressants □Mood stabilizers Anxiolytics □ Sedative-hypnotics □Cognitive enhancers | | □ | □ | □ | □ | □ |  |
| 3. Please answer information about medication use in treating the disease.  **Reference options:** □ Don’t know □Take it regularly (On time and dosage) □On dosage and not on time □On time and not on dosage □ Not taking medication | | □ | □ | □ | □ | □ |  |
| 4*. Please answer the exact times you take your medication. **Reference options:** □ Don't know □Morning fasting □ Taking before meal □Taking during meal □Taking after meal □Taking before bedtime | | □ | □ | □ | □ | □ |  |
| 5*. Please answer the total duration for which you should continue taking your current psychiatric medication.  **Reference options:** □ Don't know □ Continuous medication, 1-3 years for first episode □ Continuous medication, 3-5 years for single relapse □ Long-term medication for more than two relapses within 5 years □ Long-term treatment for significant residual symptoms | | □ | □ | □ | □ | □ |  |
| 6. Please answer your follow-up appointments after taking psychotropic medication.  **Reference options:** □ Don't know □ No need for follow-up □ Strictly adherence to doctor's orders □ Early or delayed follow-up according to your condition □ Follow-up according to your will | | □ | □ | □ | □ | □ |  |
| 7*. Please answer routine examinations that must be completed regularly while on medication. Reference options: □ Don't know □ Laboratory tests (blood counts, liver and kidney functions, etc.) □ Electrocardiogram □ B-scan ultrasonography □ Electroencephalography □Psychological counselling and observation | | □ | □ | □ | □ | □ |  |
| 8*. Please answer the common side effects of the psychotropic medication currently taking. Reference options: □ Don't know □ General common adverse reactions (gastrointestinal symptoms, cardiovascular system symptoms) □ extra vertebral system reactions (acute dystonia, Parkinson's-like disease, inability to sit still, and delayed dyskinesia) □ Metabolic syndrome (weight gain, hyperglycemia, hyperlipidemia, and hypertension) □ Disorders of the endocrine system (increased prolactin, menstrual disorders, and abnormalities of sexual function) □ Abnormalities of liver and kidney function □ Excessive Sedation, insomnia, irritability □ Lithium toxicity □ Leukopenia | | □ | □ | □ | □ | □ |  |
| 9*. Please answer the precautions you should take while using psychotropic medication.  **Reference options:** □ Don't know □ Focus on medication specifications and expiration dates □ Understand drug interactions □ Be aware of adverse drug reactions □Understand medication indications, contraindications, and warnings □ Knowing the special medication population (pregnant women, children, elderly, etc.) | | □ | □ | □ | □ | □ |  |
| **Please identify the items that need to be added and provide clear reasons for your suggestions.** |  | | | | | | |
| 10. Whether information about psychotropic medication is obtained from books, magazines or the Internet. | | □ | □ | □ | □ | □ |  |
| 11. Whether information about psychotropic medication is obtained from healthcare, loved ones, friends, or peers. | | □ | □ | □ | □ | □ |  |
| 12. Whether information about psychotropic medication is obtained from health education talks. | | □ | □ | □ | □ | □ |  |
| 13. Whether information about current psychotropic medication taken is obtained from healthcare (precautions, risks and benefits, changes in current medications, etc.). | | □ | □ | □ | □ | □ |  |
| 14. Whether adverse reactions or side effects from current psychotropic medication will be reported to the healthcare provider. | | □ | □ | □ | □ | □ |  |
| 15. Whether the current psychotropic medication is discussed in peer support or intervention groups. | | □ | □ | □ | □ | □ |  |
| **Please identify the items that need to be added and provide clear reasons for your suggestions.** |  | | | | | | |
| 16. Please answer the typical time it takes for your current psychotropic medication to take effect after administration.  **Reference options:** □ Don't know □ Within 24 hours □ Within 1 week □ 2-3 weeks □ After one month | | □ | □ | □ | □ | □ |  |
| 17*. Please answer the behaviours that may affect medication effectiveness during treatment.  **Reference options:** □ Don't know □ Switched to a different manufacturer for the same medication □ Changed medication type □ Changed medication timing □ Adjusted medication dosage □ Adjusted medication frequency □ Stopped taking medication □ Irregular lifestyle or routine □ Smoking, alcohol, strong tea, or coffee, etc. | | □ | □ | □ | □ | □ |  |
| 18. Please answer what should you do if you miss a dose of your medication?  **Reference options:** □ Don't know □ Take double dose with next dose □ Skip the missed dose and take the next dose as scheduled □ Time between next dose of medication □ Consult the doctor | | □ | □ | □ | □ | □ |  |
| 19. Please answer what actions you usually take if you accidentally take the wrong dose or an overdose of your medication?  **Reference options:** □ Don't know □ Ignore □ Attempt to accelerate metabolism (e.g., self-induced vomiting) □ Consult doctor immediately | | □ | □ | □ | □ | □ |  |
| 20. Please answer what you do if you experience a side effect from your medication?  **Reference options:** □ Don't know □ Ignore □ Take action yourself. □ Consult doctor immediately | | □ | □ | □ | □ | □ |  |
| 21. Please answer what you do if your condition changes while taking your medication?  **Reference options:** □ Don't know □ Self-discontinuation of medication □ Keep taking the medicine □ Consult doctor immediately | | □ | □ | □ | □ | □ |  |
| 22. Please answer what to do if you feel that your medication is ineffective?  **Reference options:** □ Don't know □ Self-discontinuation of medication □ Keep taking the medicine □ Consult doctor immediately | | □ | □ | □ | □ | □ |  |
| 23*. Please answer the indications for discontinuing your current psychotropic medication.  **Reference options:** □ Don't know □ Discontinue when symptoms disappear □ Discontinue if perceived as ineffective □ For the first episode, taper off medication after the maintenance phase □ For recurrent cases, taper off if the condition remains stable for over 3 years with no significant fluctuations □ Discontinue if serious drug-related adverse effects occur (e.g., malignant syndrome, myocarditis, agranulocytosis) | | □ | □ | □ | □ | □ |  |
| 24. Please answer the potential risks of interrupting your medication during treatment.  **Reference options:** □ Don't know □ No impact □ Relapse or worsening of mental illness □Prolongation of disease duration | | □ | □ | □ | □ | □ |  |
| 25. How do you typically handle situations when your treatment conflicts with your personal preferences for taking medication?  **Reference options:** □ Don't know □ Take medication according to personal preference □ Follow the prescribed medication □Active participation in medication decisions | | □ | □ | □ | □ | □ |  |
| 26. Please answer the most critical points to consider when assessing the efficacy of psychotropic medication.  **Reference options:** □ Don't know □ Psychiatric Symptoms □ Clarify Physical □ Psychological Conditions □ Physiological Conditions □ Social Function | | □ | □ | □ | □ | □ |  |
| **Please identify the items that need to be added and provide clear reasons for your suggestions.** |  | | | | | | |
| 27. Please convert gram (g) to milligrams (mg) on the medication packaging (e.g., 1g =? mg; 0.1g =? mg). | | □ | □ | □ | □ | □ |  |
| 28. Please calculate the number of tablets required for the prescribed dosage each time. | | □ | □ | □ | □ | □ |  |
| 29. Please calculate the number of tablets required for the prescribed dosage each time. | | □ | □ | □ | □ | □ |  |
| 30. Please state the expiration date of your current psychotropic medication. | | □ | □ | □ | □ | □ |  |
| 31. Please calculate the date of your next follow-up appointment or treatment. | | □ | □ | □ | □ | □ |  |
| **Please identify the items that need to be added and provide clear reasons for your suggestions.** |  | | | | | | |

**Note:** *, Indicates multiple-choice questions.

**Supplementary etable 3**  **Details of expert opinions during two rounds of Delphi**

| **Experts** **Recommendation** | **Accept or not** |
| --- | --- |
| **First round** | |
| Addition of item: Are you taking your psychotropic medications as prescribed by your doctor? | Accept |
| Addition of item: Calculate how long your current supply of psychotropic medication will last | Accept |
| Overall modification: Replace ‘Please answer’ at the start of the question with ‘You’ | Accept |
| Modification of item 4: Your current medication status | Accept |
| Advice on item 4 scoring: Define regularity as taking over 80% of the prescribed medication dose | Accept |
| Addition of option to item 6: Those with significant residual symptoms should receive long-term treatment | Accept |
| Modification of item 8: You will need to undergo routine examinations related to your mental disorders while taking your medication | Accept |
| Modification of item 8 option: Psychometric tests | Accept |
| Addition of option to item 9: Other, please list | Accept |
| Addition of option to item 10: Other, please list | Accept |
| Modification of item 10 option: Concerns about medication dosage | Accept |
| Modification of items 11-16 option: Always, often, sometimes, rarely, never | Accept |
| Modification of item 12: Your access to information about psychotropic medication from a loved one or friend | Accept |
| Modification of item 14: You inquire with your healthcare providers about information regarding your current psychotropic medication, including precautions for adverse reactions, risks and benefits, and methods for adjusting your dosage | Accept |
| Modification of item 17: How long does it typically take for your current psychotropic medication to become effective after you take it | Accept |
| Modification of item 18: Factors that may influence the effectiveness of psychotropic medication while taking the medication | Accept |
| Addition of option to item 18: Other, please list | Accept |
| Modification of items 20-22: When you… | Accept |
| Modification of item 21 option: Consult a psychiatrist immediately | Accept |
| Modification of items 21-22 option: Adhere to the prescribed medication dosage | Accept |
| Addition of option to items 21-22: Self-adjustment of dosage of medication | Accept |
| Modification of item 24 option: Stop the medication if you experience a serious adverse reaction | Accept |
| Modification of item 25 option: Withdrawal syndrome (dizziness, pain, inexplicable discomfort, anxiety, tachycardia, etc.), affecting the recovery effect, increasing the cost of treatment | Accept |
| Addition of option to item 26: Other, please list | Accept |
| Addition of option to item 27: Other, please list | Accept |
| Modification of item 31: Current frequency/number of daily doses of psychotropic medication | Accept |
| Modification of item 32: Expiration date of your current psychotropic medication | Accept |
| Modification of item 33: Date of your next follow-up appointment with your doctor | Accept |
| **Second round** | |
| Addition of item: The maximum daily dose of your current medication | Accept |
| Addition of item: The poisoning dose of your current medication | Accept |
| Modification of item 1: You are taking your psychotropic medication as prescribed by your doctor | Accept |
| Modification of item 3: What effects does the psychotropic medication you are currently taking have | Accept |
| Modification of item 4: Are you taking your medication on time and at the prescribed dosage | Not accepted |
| Modification of item 5: The specific time of day you take your medication | Accept |
| Modification of item 8: You will need to undergo routine examinations related to your regular use of psychotropic medication | Accept |
| Modification of item 8 option: Neuropsychological evaluation | Accept |
| Advice on item 9 scoring: If the patient selects ‘other,’ the score will be used to determine whether the side effect is attributable to the medication | Accept |
| Modification of item 11: Your access to information about psychotropic medication from public sources, such as books or the internet | Accept |
| Modification of item13: Your access to information about psychotropic medication from health education lectures held in hospitals or the community | Accept |
| Modification of item 18: What factors can affect the efficacy of the medication while you are taking it? | Accept |
| Modification of item 19: What should you do if you miss a dose of your medication? | Accept |
| Addition of item 20 option: Other, please list | Accept |
| Modification of item 21 option: The side effects of psychotropic medication are persistent; therefore, option content includes measures to cope with side effects and patient attitudes towards side effects | Accept |
| Addition of item 21option: Other, please list | Accept |
| Modification of items 21-22: Expressed as ‘general how to deal with’ | Accept |
| Addition of items 22-23 option: Other, please list | Accept |
| Modification of item 26: “How do you usually deal with conflicts between your doctor’s prescription and your own preferences regarding medication during treatment?” | Accept |
| Modification of item 26 option: Actively discuss medication options with your doctor | Accept |
| Modification of item 27 option: clarify physical vs. physiological conditions and psychiatric symptoms vs. psychological conditions. | Accept |
| Modification of item 30: Number of daily doses of your current psychotropic medication | Accept |
