# Supplementary material for: Development of a medication literacy assessment scale for patients with mental disorders in recovery in China: a mixed-methods Delphi study
Source: Front Psychiatry. 2025 May 27;16:1551160. doi: 10.3389/fpsyt.2025.1551160 (PMC12148901; doi:10.3389/fpsyt.2025.1551160)
Supplement: Supplementary file 2 [file Table2.docx]

**CREDES checklist items. Selection of experts, definition of consensus and quality of reporting ^[1]^**

| **Transparency and quality of reporting** | | |
| --- | --- | --- |
| **Item** | **Manuscript Section** | **Page No.** |
| Purpose well defined | Introduction | 2 |
| Rationale for Delphi Selection of experts clearly justified | Methods | 4 |
| Clear description of methods | Methods | 4-5 |
| Flow chart | Methods | 5, Figure 1 |
| Clear definition of consensus | Methods | 5 |
| Pilot test of instruments | Methods | 4 |
| Data analysis clearly justified and reported | Methods | 5 |
| Information of rounds | Methods | 4-5 |
| Transparent reporting of results | Results | 9-11 |
| Discussion of limitations | Discussion | 15 |
| Adequacy of conclusions | Discussion，Conclusions | 15 |

| **Selection criteria expert panel** |  |
| --- | --- |
| Member of organisation |  |
| Recognised authority | 🗸 |
| Relevant clinical academic expertise | 🗸 |
| Geographical scope | 🗸 |
| Setting/work field | 🗸 |
| Profession/ stakeholder | 🗸 |

1. Jünger, S., S.A. Payne, J. Brine, L. Radbruch, and S.G. Brearley, *Guidance on Conducting and REporting DElphi Studies (CREDES) in palliative care: Recommendations based on a methodological systematic review.* Palliative medicine, 2017. **31**(8): p. 684-706.
