# Supplementary material for: Development of a medication literacy assessment scale for patients with mental disorders in recovery in China: a mixed-methods Delphi study
Source: Front Psychiatry. 2025 May 27;16:1551160. doi: 10.3389/fpsyt.2025.1551160 (PMC12148901; doi:10.3389/fpsyt.2025.1551160)
Supplement: Supplementary file 3 [file Table3.docx]

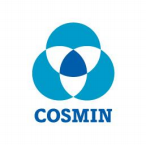
**COSMIN box 1. Standards for evaluating the quality of PROM development**

Part 1a (PROM design) and part 1b (Cognitive interview study or other pilot test) both need to be completed for each PROM because all standards of part 1a and part 1b will be included in the final rating of the quality of the PROM development. However, if a cognitive interview study or other pilot test was not performed, only the first standard in part 1b needs to be completed and the rest of the box can be skipped.

For rating the standards, the “worst score counts” method is used. A total rating for the box can be obtained by taking the lowest rating of any standard in the box. It is also possible to obtain total ratings for different parts of the boxes by taking the lowest rating of any standard of that part of the box.

| **Box 1. PROM development**  **1a. PROM design** | | | | | | |
| --- | --- | --- | --- | --- | --- | --- |
| *General design requirements* | **Very good** | **Adequate** | **Doubtful** | **Inadequate** | **Not**  **applicable** | **Results**  **on page No** |
| 1. Is a clear description provided of the construct to be measured? | Construct clearly  described |  |  | Construct not  clearly described |  | Very good  No.11 |
| 2. Is the origin of the construct clear: was a theory, conceptual framework or disease model used or clear rationale provided to define the construct to be measured? | Origin of the  construct clear |  | Origin of the  construct not clear |  |  | Very good  No.3-4 |
| 3. Is a clear description provided of the target population for which the PROM was developed? | Target  population clearly described |  |  | Target population not clearly  described |  | Very good  No.6 |
| 4. Is a clear description provided of the context of use | Context of use clearly described |  | Context of use not clearly described |  |  | Very good  No.4-5 |
| 5. Was the PROM development study performed in a sample representing the target population for which the PROM was developed? | Study performed in a sample representing the target population | Assumable that the study was performed in a sample representing the target population, but not clearly described | Doubtful whether the study was performed in a sample representing the target population | Study not performed in a sample representing the target population (SKIP standards 6-12) |  | Very good  No.6 |

| *Concept elicitation (relevance and comprehensiveness)*  6. Was an appropriate qualitative data collection method used to identify relevant items for a new PROM? | Widely recognized or well justified qualitative method used, suitable for the construct and study population | Assumable that the qualitative method was appropriate and suitable for the construct and study population, but not clearly described | Only quantitative (survey) method(s) used or doubtful whether the method was suitable for the construct and study population | Method used not  appropriate or  not suitable for  the construct or  study population |  | Very good  No.5 |
| --- | --- | --- | --- | --- | --- | --- |
| 7. Were skilled group moderators/interviewers used? | Skilled group  moderators/  interviewers  used | Group  moderators  /Interviewers had limited Experience or were trained specifically for the study | Not clear if group moderators/interviewers were trained or group moderators/interviewers not  trained and no experience |  | Not  applicable | Very good  No.6 |
| 8. Were the group meetings or interviews based on an appropriate topic or interview guide? | Appropriate  topic or  interview guide | Assumable that  the topic or  interview guide  was appropriate,  but not clearly  described | Not clear if a  topic guide was  used or doubtful  if topic or  interview guide  was appropriate  or no guide |  | Not  applicable | Very good  No.7 |
| 9. Were the group meetings or interviews recorded and transcribed verbatim? | All group meetings or interviews were recorded and transcribed verbatim | Assumable that all group meetings or interviews were recorded and transcribed verbatim, but not clearly described | Not clear if all group meetings of interviews were recorded and transcribed verbatim or recordings not transcribed verbatim or only notes were made during the group meetings/ interviews | No recording and no notes | Not applicable | Very good  No.7 |
| 10. Was an appropriate approach used to analyze the data? | A widely  recognized or  well justified  approach was  used | Assumable that  the approach  was appropriate,  but not clearly  described | Not clear what approach was used or doubtful whether the approach was appropriate | Approach not  appropriate |  | Very good  No.7 |

| 11. Was at least part of the data coded independently? | At least 50% of the data was coded by at least two researchers independently | 11-49% of the data was coded by at least two researchers independently | Doubtful if two researchers were involved in the coding or only 1- 10% of the data was coded by at least two researchers independently | Only one researcher was involved in coding or no coding | Not applicable | Very good  No.7 |
| --- | --- | --- | --- | --- | --- | --- |
| 12 Was data collection continued until saturation was reached? | Evidence provided that saturation was reached | Assumable that saturation was reached | Doubtful whether saturation was reached | Evidence suggests that saturation was not reached | Not applicable | Very good  No.11 |
| 13. For quantitative studies (surveys): was the sample size appropriate? | ≥100 | 50-99 | 30-49 | <30 | Not  applicable | Not  applicable |

| **1b. Cognitive interview study or other pilot test** | | | | | | | | |
| --- | --- | --- | --- | --- | --- | --- | --- | --- |
|  | **Very good** | **Adequate** | | **Doubtful** | **Inadequate** | | **Not applicable** | **Results**  **on page No** |
| 14. Was a cognitive interview study or other pilot test conducted? | YES | |  |  | | NO (SKIP standards 15-34) |  | Inadequate |
| *General design requirements*  15. Was the cognitive interview study or other pilot test performed in a sample representing the target population? | Study performed in a sample representing the target population | | Assumable that the study was performed in a sample representing the target population, but not clearly described | Doubtful whether the study was performed in a sample representing the target population | | Study not performed in a sample representing the target population |  | SKIP |
| *Comprehensibility* | YES | |  | Not clear (SKIP standards 17- 25) | | NO (SKIP standards 17- 25) |  | SKIP |
| 16. Were patients asked about the comprehensibility of the PROM? |  |  |  |  |  |  |  |  |

| 17. Were all items tested in their final form? | All items were tested in their final form | Assumable that all items were tested in their final form, but not clearly described | Not clear if all items were tested in their final form | Items were not tested in their final form or items were not re-tested after substantial adjustments |  | SKIP |
| --- | --- | --- | --- | --- | --- | --- |
| 18. Was an appropriate qualitative method used to assess the comprehensibility of the PROM instructions, items, response options, and recall period? | Widely recognized or well justified qualitative method used | Assumable that the method was appropriate but not clearly described | Only quantitative (survey) method(s) used or doubtful whether the method was appropriate or not clear if patients were asked about the comprehensibility of all items, response options instructions, and recall period or patients not asked about the comprehensibility of the PROM instructions or the recall period | Method used not appropriate or patients not asked about the comprehensibility of all items and response options |  | SKIP |
| 19. Was each item tested in an appropriate number of patients?  For qualitative studies | ≥7  ≥50 | 4-6  ≥30 | <4 or not clear  <30 or not clear |  |  | SKIP |
| For quantitative (survey) studies |  |  |  |  |  |  |
| 20. Were skilled interviewers used? | Skilled group moderators/ interviewers used | Group moderators /interviewers had limited experience or were trained specifically for the study | Not clear if group moderators /interviewers were trained or group moderators /interviewers not trained and no experience |  | Not applicable | SKIP |
| 21. Were the interviews based on an appropriate interview guide? | Appropriate topic or interview guide | Assumable that the topic or interview guide was appropriate, but not clearly described | Not clear if a topic guide was used or doubtful if topic or interview guide was appropriate or no guide |  | Not applicable | SKIP |
| 22. Were the interviews recorded and transcribed verbatim? | All group meetings or interviews were recorded and transcribed verbatim | Assumable that all group meetings or interviews were recorded and transcribed verbatim, but not clearly described | Not clear if all group meetings or interviews were recorded and transcribed verbatim or recordings not transcribed verbatim or only notes were made during the group meetings/ interviews | No recording and no notes | Not applicable | SKIP |
| 23. Was an appropriate approach used to analyze the data? | A widely recognized or well justified approach was used | Assumable that the approach was appropriate, but not clearly described | Not clear what approach was used or doubtful whether the approach was appropriate | Approach not appropriate |  | SKIP |
| 24. Were at least two researchers involved in the analysis? | At least two researchers involved in the analysis | Assumable that at least two researchers were involved in the analysis, but not clearly described | Not clear if two researchers were included in the analysis or only one researcher involved in the analysis |  |  | SKIP |
| 25. Were problems regarding the comprehensibility of the PROM instructions, items, response options, and recall period appropriately addressed by adapting the PROM? | No problems found or problems appropriately addressed and PROM was adapted and retested if necessary | Assumable that there were no problems or that problems were appropriately addressed, but not clearly described | Not clear if there were problems or doubtful if problems were appropriately addressed | Problems not appropriately addressed or PROM was adapted but items were not re-tested after substantial adjustments. | Not applicable | SKIP |
| *Comprehensiveness* | YES |  | NO or not clear (SKIP standards 27-35) |  |  | SKIP |
| 26. Were patients asked about the comprehensiveness of the PROM? |  |  |  |  |  |  |
| 27. Was the final set of items tested? | The final set of items was tested | Assumable that the final set of items was tested, but not clearly described | Not clear if the final set of items was tested or not the final set of items was tested or the set of items was not retested after items were removed or added |  |  |  |
| 28. Was an appropriate method used for assessing the comprehensiveness of the PROM? | Widely recognized or well justified method used | Assumable that the method was appropriate but not clearly described or only quantitative (survey) method(s) used | Doubtful whether the method was appropriate or method used not appropriate |  |  | SKIP |
| 29. Was each item tested in an appropriate number of patients?  For qualitative studies  For quantitative (survey) studies | ≥ 7  ≥ 5 0 | 4 - 6  ≥ 30 | < 4 or not clear  < 30 or not clear |  |  | SKIP |
| 30. Were skilled interviewers used? | Skilled  interviewers used | Interviewers had limited  experience or were trained specifically for the study | Not clear if  interviewers were trained or  interviewers not trained and no experience |  | Not  applicable | SKIP |
| 31. Were the interviews based on an appropriate interview guide? | Appropriate  topic or  interview guide | Assumable that the topic or  interview guide was appropriate, but not clearly  described | Not clear if a  topic guide was used or doubtful if topic or  interview guide was appropriate or no guide |  | Not  applicable | SKIP |
| 32. Were the interviews recorded and transcribed verbatim? | All group  meetings or  interviews were recorded and  transcribed verbatim | Assumable that all group  meetings or  interviews were recorded and  transcribed  verbatim, but not clearly described | Not clear if all  group meetings or interviews  were recorded  and transcribed verbatim or  recordings not  transcribed  verbatim or only notes were made during the group meetings/  interviews or no recording and no notes |  | Not  applicable | SKIP |
| 33. Was an appropriate approach used to analyze the data? | A widely  recognized or well justified approach was used | Assumable that the approach  was appropriate, but not clearly  described | Not clear what approach was  used or doubtful whether the  approach was appropriate or approach not appropriate |  |  | SKIP |
| 34. Were at least two researchers involved in the analysis? | At least two  researchers  involved in the analysis | Assumable that at least two  researchers were involved in the  analysis, but not clearly described | Not clear if two  researchers were included in the  analysis or only one researcher involved in the analysis |  |  | SKIP |
| 35. Were problems regarding the comprehensiveness of the PROM appropriately addressed by adapting the PROM? | No problems  found or  problems  appropriately  addressed and PROM was  adapted and re- tested if  necessary | Assumable that there were no  problems or that problems were appropriately  addressed, but not clearly  described | Not clear if there were problems or doubtful if  problems were appropriately addressed or PROM was  adapted but  items were not re-tested after substantial  adjustments | Problems not  appropriately addressed | Not  applicable | Not  applicable |
