# Supplementary material for: Development of a medication literacy assessment scale for patients with mental disorders in recovery in China: a mixed-methods Delphi study
Source: Front Psychiatry. 2025 May 27;16:1551160. doi: 10.3389/fpsyt.2025.1551160 (PMC12148901; doi:10.3389/fpsyt.2025.1551160)
Supplement: Supplementary file 4 [file Table4.docx]

**Supplementary** **Material 4**  Characteristics of the included studies

| **Author year** | **Study location** | **Aim of study** | **Study design** | **Sample** | **Construct definition (paraphrase)** | **Dimension/Themes** |
| --- | --- | --- | --- | --- | --- | --- |
| Qin et al.^[1]^ (2022) | China | Revise the medication literacy assessment scale for hypertension patients and conduct reliability and validity testing to provide a scientific and convenient evaluation tool | Psychometric study | Hypertension patients | Medication literacy: The ability of hypertensive patients to obtain, understand, and evaluate hypertensive disease and drug information to make appropriate medication decisions and take medication-related actions. | Knowledge; Attitude; Skill; Practice. |
| Zhao Xue ^[2]^(2023) | China | Under the guidance of the medication literacy theory, develop and evaluate the reliability and validity of a medication literacy scale for older patients with chronic diseases, making it an effective measurement tool. | Psychometric study | Older patients with chronic diseases | Medication literacy: The ability of individuals to acquire, understand, and evaluate medication-related information about diseases, and to use this information to make correct medication decisions and behaviors. | Information acquisition ability; Medication knowledge reserve; Communication and interaction ability; Critical ability. |
| Zheng et al.^[3]^ (2016) | China | To translate and revise the Medication Literacy Assessment in English (Med Lit Rx SE English) and evaluate its validity and reliability. | Psychometric study | Non-hospitalized patients | Medication literacy: The ability of individuals to acquire, accurately understand, and correctly handle basic medication information, and make appropriate decisions. | Four case situations: injectable medication; Paediatric dose of non-prescription medication for fever, tablets; Active ingredients on a non-prescription product |
| Zhou et al. ^[4]^(2022) | China | Construct a medication literacy assessment scale for caregivers of patients with schizophrenia, providing a professional approach for evaluating the medication literacy of caregivers of patients with schizophrenia. | Psychometric study | Caregivers of patients with schizophrenia | Caregiver medication literacy: The ability of caregivers to acquire, understand, and evaluate medication-related information about the patient’s condition, and to use this information to make correct medication decisions and behaviors in order to maintain the patient’s health. | Information and knowledge; Cognition and attitude; Passive involvement; Active involvement. |
| ^[5]^Zhou et al. (2022) | China | To develop a medication literacy scale for parents of children with epilepsy that is suitable for China’s cultural context, and to conduct reliability and validity testing, providing an effective measurement tool for healthcare professionals. | Psychometric study | Parents of children with epilepsy | Medication literacy: The ability of parents of children with epilepsy to obtain, understand, communicate, calculate, and process information about antiepileptic drugs, in order to make informed medication decisions for the child's disease treatment, ensuring the safe and effective use of antiepileptic drugs. | Disease and medication knowledge; Medication beliefs; Medication abilities; Medication behaviors. |
| Gentizon et al.^[6]^ (2022) | Switzerland | to conceptualize and evaluate the content validity of a new medication literacy measure, the medication Literacy Assessment of Geriatric patients and informal caregivers (the MED-fLAG), including the preliminary acceptability and feasibility of its use during a hospital stay. | Psychometric study | Older patients and informal caregivers | medication literacy: Abilities to access, comprehend and interact with medication-related information | Functional medication literacy; Interactive medication literacy; Critical medication literacy. |
| Gnägi et al.^[7]^ (2022) | Switzerland | To develop a consensus-based instrument [MELIA] to assess the medication literacy of older home care patients to ultimately optimize medication safety. | Psychometric study | Older home care patients | Medication literacy: The degree to which the patient has the specific information about his or her medication that is necessary to make informed decisions and execute the necessary actions | Questions for patients on their taking and understanding of their medications; Questions to ask the patient about using aids and obtaining medications; Questions for the patient on medication taking; Questions about the patient’s motor and visual capacities |
| Horvat et al.^[8]^ (2020) | Slovenia | To develop and validate a self-administered performance-based questionnaire measuring functional medication literacy and to evaluate functional medication literacy among the Slovenian general population. | Psychometric study | Slovenian general population | Medication literacy: The ability of individuals to safely and appropriately access, understand and act on basic medication information. | Functional medication literacy (dosage, adverse effects, interactions, precautions, and other information.) |
| King et al.^[9]^ (2011) | America | To conceptualize the Pharmacotherapy Literacy construct. | Cross-sectional study | Generic | Pharmacotherapy Literacy: An individual’s capacity to obtain, evaluate, calculate, and comprehend basic information about pharmacotherapy and pharmacy related services necessary to make appropriate medication-related decisions, regardless of the mode of content delivery (e.g. written, oral, visual images and symbols). | Not applicable |
| Lin et al.^[10]^ (2020) | China | To describe the comprehensive development process of this instrument, including the conceptual framework, process of item development and reduction, scoring and application, and to share the instrument, its short form, as well as the English version for further use in real world practice or research. | Cross-sectional study | All patients | medication literacy: The ability of individuals to safely and appropriately acquire, understand, and act upon basic medication information. | Vocabulary of medications; OTC labels; Prescription (Rx) labels; Dietary supplement (DS) commercial advertisements. |
| Miner et al.^[11]^ (2018) | American | To explore medication literacy in a group of Somali older adults and their families using qualitative secondary analysis. | Qualitative methodology | Somali older adults receiving home care | Medication literacy: The ability of individuals to access and understand medication information and then use that information to act and take their medication in a safe and appropriate way. | (1) Medication literacy is needed among Somali older adults and their families; (2) Using home healthcare to improve medication literacy; (3) Better communication is essential to improving medication literacy; (4) Medication literacy is an intersecting family and social issue. |
| Neiva et al.^[12]^ (2022) | Brazilian | To propose a conceptual model to support the development of an instrument to measure medication literacy. | Review | Generic | Medication literacy: an individual’s ability to access, understand, communicate, and evaluate information about medications, as well as perform essential calculations for proper medication use. It can be seen as an interaction between the demands of the social environment, including the healthcare system, and an individual's competencies to use medications correctly. | Functional literacy; Communicative literacy; Critical literacy; Numeracy. |
| Neiva et al.^[13]^ (2023) | Brazilian | To develop and evaluate the content validity of an instrument to measure medication literacy in older adults. | Psychometric study | Older adults | Medication literacy: an individual’s ability to safely and appropriately access, understand, and act on medication information. | Dimensions-functional literacy; communicative literacy; critical literacy, numeracy.  sub-dimensions-comprehend; communicate; access; evaluate; calculate. |
| Pouliot et al.^[14]^ (2018) | Canada | To reach consensus on the statements and a definition of medication literacy. | Delphi study | Generic | Medication literacy: individuals can obtain, comprehend, communicate, calculate and process patient-specific information about their medications to make informed medication and health decisions in order to safely and effectively use their medications, regardless of the mode by which the content is delivered (e.g. written, oral and visual). | (1) the type of information necessary for optimal and safe use of medication; (2) the skills necessary for optimal and safe use of medication; (3) the format of information and pharmacy services required; (4) the outcomes and goals of medication literacy. |
| Sauceda et al.^[15]^ (2012) | American | To evaluate the psychometric properties of a newly developed Medication Literacy Assessment in Spanish and English (Med Lit Rx SE) | Psychometric study | General population in non-clinical settings | Medication literacy: The ability of individuals to safely and appropriately access, understand and act on basic medication information. | Document Literacy; Numeracy; Prose Literacy. |
| Stilley et al.^[16]^ (2014) | American | To details development and psychometric properties of a health literacy measure for oral medications based on design of the Newest Vital Signs. | Psychometric study | Adult liver transplant recipients | Health literacy: The degree to which individuals have the capacity to obtain, process, and understand basic health information and services needed to make appropriate health decisions. | Prose; Numeracy; Documentation. |
| Torun et al.^[17]^ (2023) | Turkey | To develop a Turkish scale to assess medication literacy and to evaluate its psychometric properties among adults having at least 12 years of education in Türkiye. | Psychometric study | Adults | Medication literacy: The degree to which individuals can obtain, comprehend, communicate, calculate, and process patient-specific information about their medications to make informed medication and health decisions in order to safely and effectively use their medications, regardless of the mode by which the content is delivered. | Prose; Document literacy; Numeracy. |
| Ubavić et al.^[18]^ (2019) | Serbia | To develop an instrument for assessment of pharmacotherapy health literacy among parents of pre-school children in Serbia (PTHL-SR) and to evaluate psychometric properties. | Psychometric study | Parents  of pre-school children | Medication literacy: An individual’s capacity to obtain, evaluate, calculate, and comprehend basic information about pharmacotherapy and pharmacy related services necessary to make appropriate medication-related decisions, regardless of the mode of content delivery | Knowledge; Understanding; Numeracy; Access to medicine related information. |
| Yeh et al.^[19]^ (2017) | China | To develop and validate the first Chinese medication literacy measure (Ch MLM). | Psychometric study | Adults | Unclear | Vocabulary; non‐prescription drug; prescription drug; drug advertisement. |
| Zhong et al.^[20]^ (2020) | China | To develop the medication literacy scale for patients with hypertension, and to test the reliability and validity of the scale. | Psychometric study | Hypertensive patients | Medication literacy: The degree to which individuals can obtain, comprehend, communicate, calculate, and process patient-specific information about their medications to make informed medication and health decisions in order to safely and effectively use their medications, regardless of the mode by which the content is delivered. | Knowledge; Attitude; Skill; practice. |

Uncategorized References

[1] QIN N, DUAN Y, YAO Z, et al. Psychometric properties and validation of the revised Chinese Medication Literacy Scale for Hypertensive Patients (C-MLSHP-R) [J]. Frontiers in Cardiovascular Medicine, 2022, 9.

[2] Zhao X. Development of a Medication Literacy Scale for Elderly Chronic Disease Patients [D]. Lanzhou University, 2023.

[3] Zheng F, Zhong ZQ, Ding SQ, et al. Compilation and Evaluation of the Medication Literacy Assessment Scale [J]. Journal of Central South University (Medical Edition)., 2016, 41(11): 1226-31.

[4] ZHOU H. Study on development and test of the Medication LiteracyScale for Caregivers of Patients with Schizophrenia [D]. Kunming Medical University, 2022.

[5] Zhou Y. Development and Reliability and Validity Testing of the Medication Literacy Scale for Parents of Children with Epilepsy [D]. Guangdong Pharmaceutical University, 2022.

[6] GENTIZON J, FLEURY M, PILET E, et al. Conceptualization and content validation of the MEDication literacy assessment of geriatric patients and informal caregivers (MED-fLAG) [J]. J Patient Rep Outcomes, 2022, 6(1): 87.

[7] GNäGI R, ZúñIGA F, BRUNKERT T, et al. Development of a medication literacy assessment instrument (MELIA) for older people receiving home care [J]. Journal of advanced nursing, 2022, 78(12): 4210-20.

[8] HORVAT N, KOS M. Development, validation and performance of a newly designed tool to evaluate functional medication literacy in Slovenia [J]. International Journal of Clinical Pharmacy, 2020, 42(6): 1490-8.

[9] KING S R, MCCAFFREY D J, BOULDIN A S. Health literacy in the pharmacy setting: Defining pharmacotherapy literacy [J]. Pharmacy Practice, 2011, 9(4): 213-20.

[10] LIN H W, CHANG E H, KO Y, et al. Conceptualization, development and psychometric evaluations of a new medication-related health literacy instrument: The chinese medication literacy measurement [J]. International Journal of Environmental Research and Public Health, 2020, 17(19): 1-17.

[11] MINER S, MCDONALD M V, SQUIRES A. Medication Literacy and Somali Older Adults Receiving Home Care [J]. Home healthcare now, 2018, 36(5): 295-303.

[12] NEIVA PANTUZZA L L, NASCIMENTO E D, CREPALDE-RIBEIRO K, et al. Medication literacy: A conceptual model [J]. Research in social & administrative pharmacy : RSAP, 2022, 18(4): 2675-82.

[13] PANTUZZA L L N, DO NASCIMENTO E, BOTELHO S F, et al. Development and content validation of the medication literacy test for older adults (TELUMI) [J]. Arch Gerontol Geriatr, 2023, 112: 105027.

[14] POULIOT A, VAILLANCOURT R, STACEY D, et al. Defining and identifying concepts of medication literacy: An international perspective [J]. Research in social & administrative pharmacy : RSAP, 2018, 14(9): 797-804.

[15] SAUCEDA J A, LOYA A M, SIAS J J, et al. Medication literacy in Spanish and English: Psychometric evaluation of a new assessment tool [J]. Journal of the American Pharmacists Association, 2012, 52(6): e231-e40.

[16] STILLEY C S, TERHORST L, FLYNN W B, et al. Medication health literacy measure: development and psychometric properties [J]. Journal of nursing measurement, 2014, 22(2): 213-22.

[17] TORUN O, MEMIç İ, AY P, et al. Development and Evaluation of a Turkish Scale to Assess Medication Literacy for Adults [J]. Turkish Journal of Pharmaceutical Sciences, 2023, 20(2): 84-90.

[18] UBAVIĆ S, KRAJNOVIĆ D, BOGAVAC-STANOJEVIĆ N. Pharmacotherapy literacy questionnaire for parents of pre-school children in Serbia: Construction and psychometric characteristics [J]. Vojnosanitetski Pregled, 2019, 76(10): 1054-61.

[19] YEH Y C, LIN H W, CHANG E H, et al. Development and validation of a Chinese medication literacy measure [J]. Health expectations : an international journal of public participation in health care and health policy, 2017, 20(6): 1296-301.

[20] ZHONG Z, SHI S, DUAN Y, et al. The Development and Psychometric Assessment of Chinese Medication Literacy Scale for Hypertensive Patients (C-MLSHP) [J]. Frontiers in Pharmacology, 2020, 11.
